# The Inhibitory Thermal Effects of Focused Ultrasound on an Identified, Single Motoneuron

**DOI:** 10.1523/ENEURO.0514-20.2021

**Published:** 2021-04-27

**Authors:** Morgan N. Collins, Wynn Legon, Karen A. Mesce

**Affiliations:** 1 Graduate Program in Neuroscience, University of Minnesota, St. Paul, MN 55108; 2 Department of Neurological Surgery, School of Medicine, University of Virginia, Charlottesville, VA 22901; 3 Departments of Entomology and Neuroscience, University of Minnesota, St. Paul, MN 55108

**Keywords:** conduction block, electrophysiology, focused ultrasound, invertebrates, motoneurons, thermal inhibition

## Abstract

Focused ultrasound (US) is an emerging neuromodulation technology that has gained much attention because of its ability to modulate, noninvasively, neuronal activity in a variety of animals, including humans. However, there has been considerable debate about exactly which types of neurons can be influenced and what underlying mechanisms are in play. Are US-evoked motor changes driven indirectly by activated mechanosensory inputs, or more directly via central interneurons or motoneurons? Although it has been shown that US can mechanically depolarize mechanosensory neurons, there are no studies that have yet tested how identified motoneurons respond directly to US and what the underlying mechanism might be. Here, we examined the effects of US on a single, identified motoneuron within a well-studied and tractable invertebrate preparation, the medicinal leech, *Hirudo verbana*. Our approach aimed to clarify single neuronal responses to US, which may be obscured in other studies whereby US is applied across a diverse population of cells. We found that US has the ability to inhibit tonic spiking activity through a predominately thermal mechanism. US-evoked effects persisted after blocking synaptic inputs, indicating that its actions were direct. Experiments also revealed that US-comparable heating blocked the axonal conduction of spontaneous action potentials. Finally, we found no evidence that US had significant mechanical effects on the neurons tested, a finding counter to prevailing views. We conclude that a non-sensory neuron can be directly inhibited via a thermal mechanism, a finding that holds promise for clinical neuromodulatory applications.

## Significance Statement

Much of the enthusiasm regarding focused ultrasound (US) neuromodulation stems from human and other mammalian noninvasive transcranial stimulation and its effects on evoked potentials or motor activity. However, there is considerable debate in the field of US neuromodulation about exactly which types of neurons can be influenced, what the direct outcomes are, and what underlying mechanisms are responsible. In our study, conducted in the medicinal leech *Hirudo verbana*, we examine for the first time whether a motoneuron could respond to US, which was accomplished at the single-cell level. We found that under conditions whereby US generated sufficient heat (2–3°C), an inhibitory response was generated. These results have important implications for the noninvasive treatment of chronic pain and other neural disorders.

## Introduction

Focused ultrasound (US) is an emerging neuromodulation technology with the potential to modulate neuronal activity noninvasively and with great precision. Although US’s effects on neural tissues have been investigated for nearly a century (Harvey, 1929), renewed interest in US has recently emerged because of the recognized therapeutic value of electrical neuromodulation technologies (Miocinovic et al., 2013; Chakravarthy et al., 2016; Grider et al., 2016). Because US can target deep neural structures noninvasively with accuracy on the order of millimeters (Anderson et al., 1951; Hynynen and Clement, 2007; Legon et al., 2018a), it could provide a viable alternative to implantable neuromodulatory devices, sparing patients the risks and financial burdens of surgery.

Despite the advantages of US, its reported effects are variable. These effects in mammalian systems range from neuronal excitation (Tyler et al., 2008; Tufail et al., 2010; Yoo et al., 2011; Kim et al., 2012, 2014, 2015; Downs et al., 2018) to inhibition (Anderson et al., 1951; Fry et al., 1958; Takagi et al., 1960; Shealy and Henneman, 1962; Rinaldi et al., 1991; Min et al., 2011; Legon et al., 2014). Invertebrate preparations including *Caenorhabditis elegans*, earthworms and crayfish share similar disparities, with reports of both neuronal excitation (Kubanek et al., 2018; Lin et al., 2019) and inhibition (Wright et al., 2017; Yoo et al., 2017; Zhou et al., 2017).

One potential factor contributing to this variability is the use, by most studies, of response measures of multiunit activity, including compound action potentials (Tsui et al., 2005; Wright et al., 2015; Yoo et al., 2017), event-related potentials (Legon et al., 2014, 2018b; Kim et al., 2015), and BOLD signals (Yoo et al., 2011; Ai et al., 2016, 2018). Population-level measurements can be difficult to interpret, as effects that appear to be direct on target tissues may result from mechanical activation of synaptically-coupled sensory neurons. Examples include auditory hair cells, which can produce widespread cortical activation following US brain application (Guo et al., 2018; Sato et al., 2018), and cells expressing ion channels sensitive to US, including members of the Piezo (Prieto et al., 2018), TRP (Yoo et al., 2020), and DEG/ENaC/ASIC (Kubanek et al., 2018) ion channel families, which are most commonly associated with sensory neurons.

In this study, we sought to determine whether single, non-sensory neurons could directly respond to US, and what the mode of action might be. To reduce cell-to-cell variability, minimize potential confounding indirect synaptic effects, and focus on a distinct class of non-sensory cells, we studied the actions of US on a single identified motoneuron, the dorsal longitudinal excitor-3 (DE-3). This neuron’s morphology and its physiological activity can be uniquely identified across multiple preparations of the medicinal leech, *Hirudo verbana*, which has an extensively well characterized and tractable central nervous system (Kristan et al., 2005). To our knowledge, ours is the first study to examine how US directly influences a single motoneuron to determine whether cell types not specialized for mechanosensory transduction would respond to US. Furthermore, our paradigm did not require the removal of nervous tissue from the animal, allowing us to examine the effects of US on a single neuron (specifically its axon) within a functional neural network, as well as avoiding alterations to intrinsic neuronal properties that may occur in culture. This single-cell approach not only enabled us to detect, with precision, whether US was an effective actuator of neuronal change, but enabled us to understand the extent to which its mechanical or thermal properties contributed to the cell’s alterations.

## Materials and Methods

### Animal preparation and recording substrates

**Figure 1.**
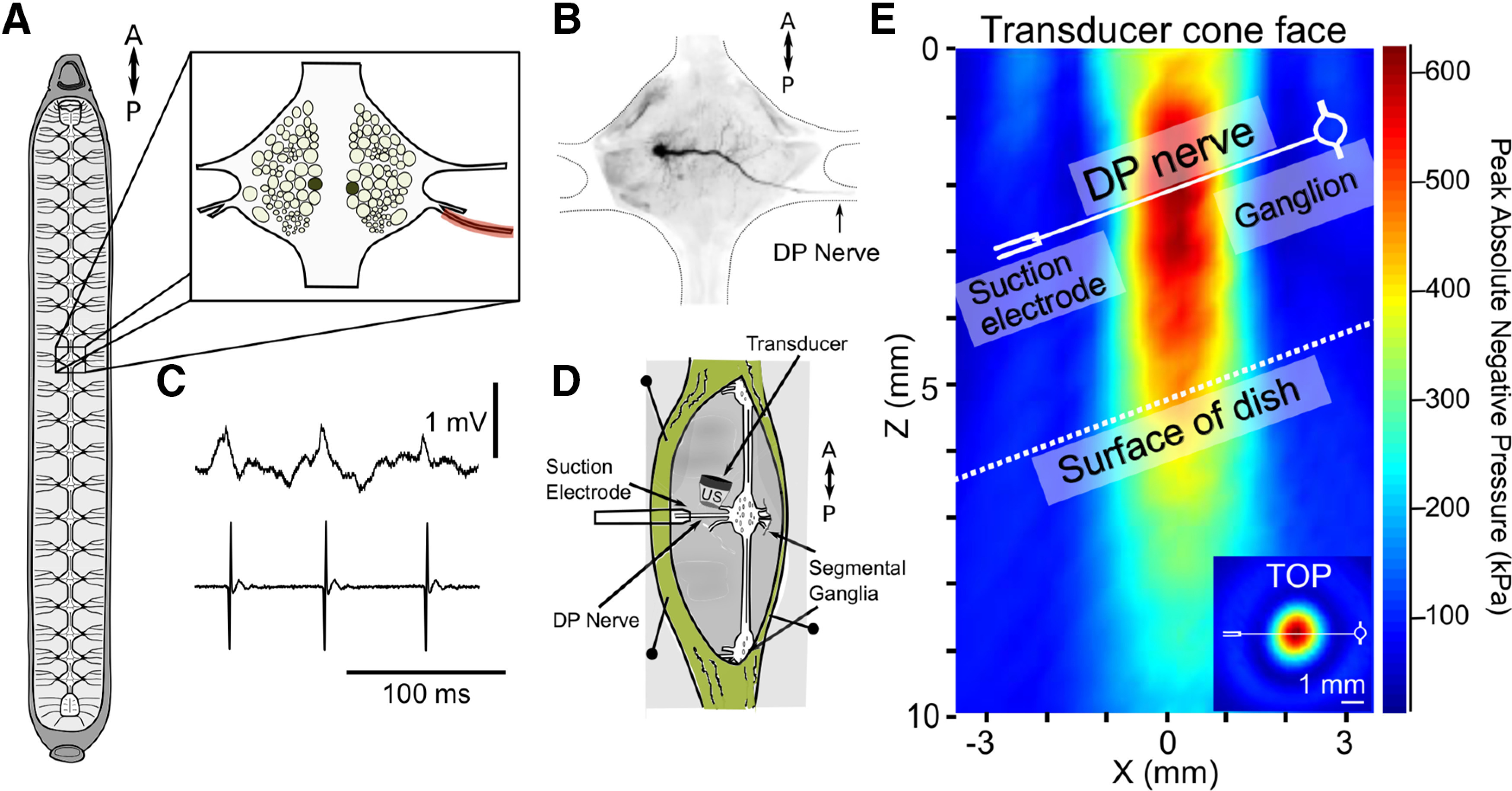
Schematic overview of the experimental preparation (medicinal leech, *H. verbana*), and details of the US transducer and its placement. ***A***, Body of the leech dissected open to reveal the CNS, consisting of a cephalic ganglion, 21 individual segmental ganglia and a posterior compound ganglion, all interconnected by longitudinal connectives. Anterior and posterior orientations are indicated by the double-headed arrow (shown throughout). One of the segmental ganglia (ganglion-10) is shown magnified, depicting the bilateral somata located on the dorsal surface of the ganglion. The two filled circles mark locations of the somata of the paired DE-3 motoneurons; one of the two DP nerves (on the right) is tinted red. ***B***, A single segmental ganglion (dorsal surface) showing the morphology of the left DE-3 motoneuron obtained by intracellular iontophoretic injection of Neurobiotin. Note: the axon exits the ganglion through the right DP nerve (arrow). A schematized outline of the ganglion and anterior and posterior nerve roots are also shown. ***C***, Dual intracellular somatic (top) and extracellular DP nerve (bottom) recordings of spontaneous spiking in the DE-3 motoneuron. ***D***, Schematic of semi-intact preparation placed in the recording chamber (not to scale) and positioning of the US transducer and suction electrode on the exposed DP nerve. Portrayal of the recording chamber is shown in an artificial upright orientation to better align with the next panel (***E***). ***E***, Pressures (in kPa) emitted from the face of the US transducer (top of graph). Pressure values (right of graph) are colorized from red (high) to blue (low). Distance from the face of the US transducer is marked in millimeters (left side of graph; 0-mm marks transducer cone face). Hydrophone data (linearly interpolated) are shown at maximum intensity in relationship with the DP nerve and top surface of the recording dish overlaid on scan. US pressures are shown in vertical (i.e., depth) and horizontal (inset) cross-section in relation to the ganglion (white circle, right) and suction electrode (white tube-shape, left). All proportions in ***E*** are depicted accurately.

Hermaphroditic adult leeches (*H. verbana*) were obtained from Niagara Medical Leeches (Niagara) and housed at room temperature (22–24°C) in a large tank filled with pond water. Leeches acclimated and maintained at room temperature can remain viable for up to a year or longer as described by others (Harley et al., 2015). Leeches were anaesthetized on ice (<5 min) before dissection. For intact preparations (all US and control trials), leeches were pinned dorsal-side-up on a porous beeswax dish; dissections were minimal and limited to exposing the targeted dorsal posterior (DP) nerve, which contained the axon of the targeted motoneuron, DE-3. An overview of the neuroanatomy of the leech, the DE-3 motoneuron’s spike profile, and experimental paradigm are shown in Figure 1.

For isolated preparations (laser, wire, and low-heat US trials), we removed a portion of the dorsal nerve cord containing three segmental ganglia with attached DP nerves. For laser and wire trials, we pinned the nerve cord dorsal-side-up on a silicone polymer surface (Sylgard, Dow Corning). Low-heat US trials were performed using a latex-bottomed dish over a 500 ml bottle filled with a large sponge and deionized, degassed water (depth = 15 cm). All preparations were bathed in normal saline during dissection, and either normal or calcium-free saline during experimental trials. Normal saline (adapted from Nicholls and Baylor, 1968) was composed of the following: 115 mm NaCl, 4.0 mm KCl, 1.8 mm CaCl_2_, 1.5 mm MgCl_2_, 10.0 mm glucose, and 10.0 mm Trizma preset crystals (pH 7.4). Calcium-free saline was prepared by replacing calcium with equimolar manganese as described (recipe from Olsen and Calabrese, 1996).

### Electrophysiology

Extracellular DE-3 activity was recorded using a suction electrode placed on the distal end of the DP nerve; suction electrodes were made in-house, and had a tip diameter of ca. 50 μm. Signals were amplified by a Model 1700 A-M Systems differential A-C amplifier, and digitized by an Axon CNS Digidata 1440A (Molecular Devices). Intracellular sharp recordings of DE-3 activity were performed using glass electrodes pulled to a resistance of 25–60 MΩ with a micropipette puller (Sutter Instrument Co, model P-87) and filled with 2 m potassium acetate. Signals were amplified by an IX2-700 dual intracellular preamp (Dagan Corp.) and digitized as previously described. All signals were recorded with the pClamp software package (Molecular Devices), and imported into MATLAB (MathWorks) for analysis. Extracellular DE-3 activity was identified as the largest spontaneously active unit in the DP recording; somatic intracellular recordings were confirmed to be DE-3 by the cell’s size and position, and the correspondence of intracellular and extracellular spikes. The rising phase of the DE-3 extracellularly recorded action potential was typically negative in our recordings; extracellular traces in all figures were inverted for more intuitive viewing. By convention, we have omitted vertical scale bars from extracellular traces because of our use of an AC-coupled amplifier.

### Experimental design and statistical analyses

All trials were performed at room temperature (22–24°C), the temperature to which the animals had acclimated. Across experiments, we used a total of 106 nerves from 41 hermaphroditic animals (approximately two to three nerves per animal). Sample sizes for heat-only paradigms were ∼10 per condition, consistent with prior leech electrophysiological studies (Puhl and Mesce, 2008; Harley et al., 2015). US and control paradigms had larger sample sizes (∼20) to enable subsets of different conditions (e.g., Ca^2+^-free vs regular salines).

We first tested DP nerves (*N* = 10) with US at five different application durations (100 ms, 316 ms, 1.0 s, 3.16 s, and 10 s). Applications shorter than 10 s failed to discernibly modulate DE-3 firing rate in any of the nerves tested or evoke firing in other neurons whose axons pass through the DP nerve. To determine whether longer durations would yield more consistent modulation, we extended the application time to 30 s. Our initial attempts suggested that this stimulus duration yielded more reliable and dramatic modulation than shorter durations.

**Figure 2.**
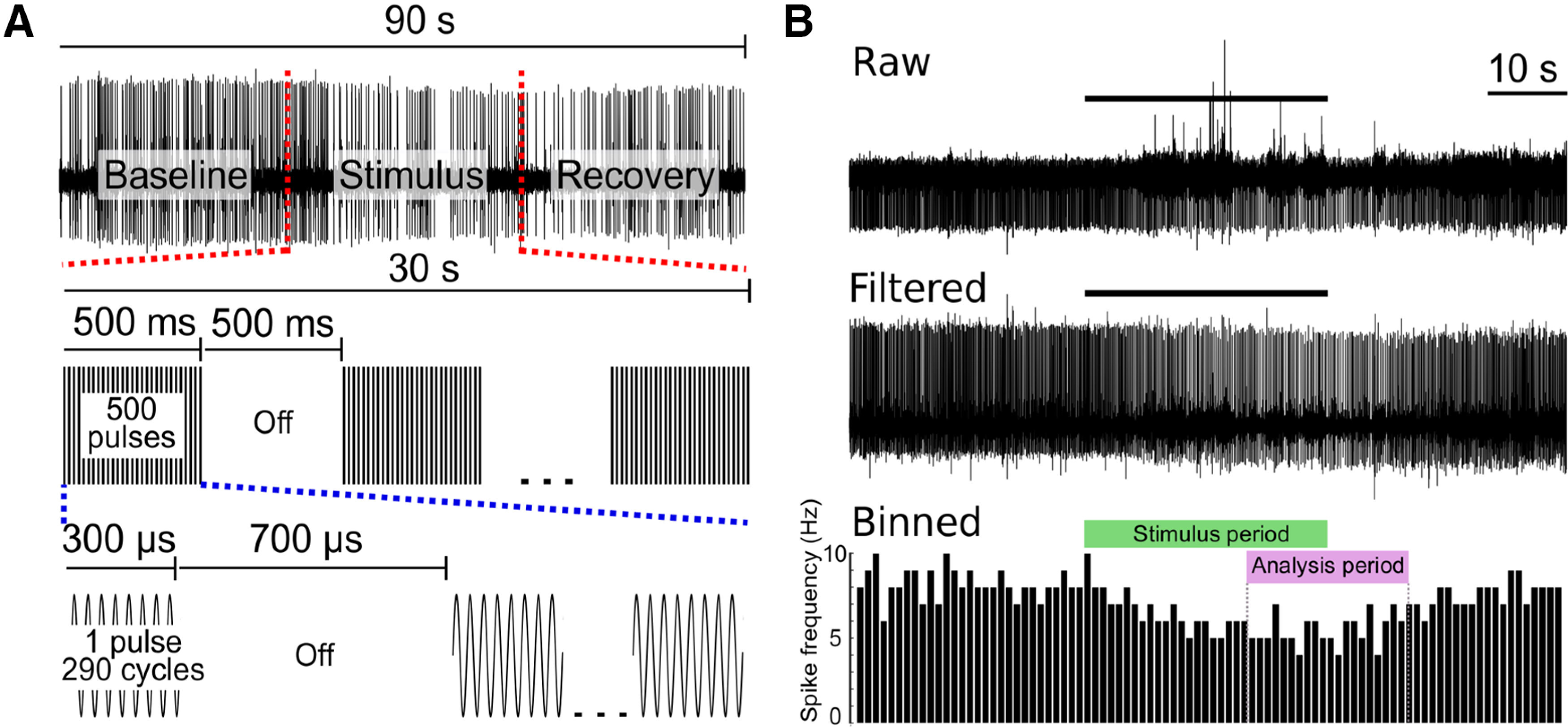
US pulse parameters and trial design with an example response. ***A***, Each US application trial lasted 90 s in duration wherein 960-kHz pulsed US was applied for 30 s, preceded by a baseline period (30 s; example, top trace). Each US pulse was 300 μs in duration (bottom trace), and was applied with a 1-kHz pulse repetition frequency with a 50% duty cycle (middle trace; intrapulse duty cycle of 30%). ***B***, Example of extracellular DE-3 recorded spikes with the US-associated artifact (top trace). The same recording after filtering the data with a sixth-order low-pass Butterworth filter (frequency cutoff = 1000 Hz; middle trace). Extracellular spike data from the filtered trace binned in 1-s intervals to yield spike frequency in Hertz (bottom trace). The US application period is denoted by the stimulus period box; the analysis period is denoted by the analysis box.

All reported trials were performed on stimulus-naive nerves. Typical trials were 90 s in duration: 30 s of baseline, 30 s of stimulus application, and 30 s of recovery (a sample trial is shown in Fig. 2*A*). Shorter pulse trials were also 90 s, but with longer recovery periods to compensate for shorter stimulation periods. Recovery periods were extended in trials in which nerves failed to return to within 20% of baseline firing rate until recovery was achieved, or until sufficient time passed to impair nerve viability (∼1 h). Control trials were equivalent in duration but did not include stimulus application.

Data acquired by pClamp software were imported into MATLAB (R2018b, MathWorks) for all analyses. DE-3 spikes were identified via manually-adjusted thresholding; larger spikes attributed to other cell types (rare) were excluded from analysis via indexing to ensure accurate frequency calculations. DE-3 spikes were binned in 1-s bins for the duration of each trial to yield frequencies in Hz (Fig. 2*B*). Response periods for US were defined as seconds 50–70 of the trial period (Fig. 2*B*), as maximal effects were observed starting 20 s into US application. For the heat-only stimuli, which yielded effects more quickly, the response period was shifted earlier to seconds 40–60 of the trial period.

**Figure 3.**
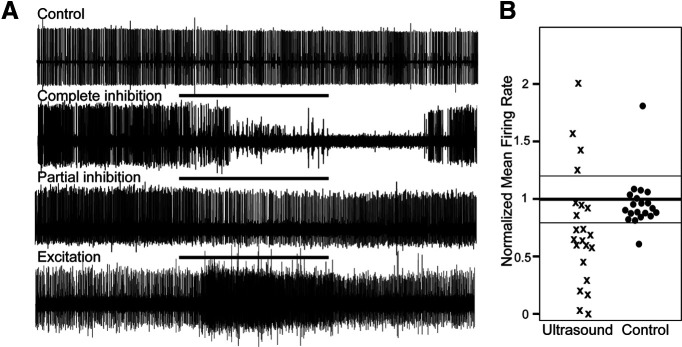
US modulates the activity of motoneuron DE-3. ***A***, Representative extracellular traces of DE-3 spiking activity in the absence of US (top trace) and in response to US applied for 30 s (horizontal lines denote US application). The predominant response was a reduction in spike activity (two middle traces), and less frequently an increase in spiking activity, sometimes with the recruitment of additional smaller and larger units (bottom trace). US-excited units larger than DE-3 can obscure the DE-3 spike (which is the largest spontaneous unit in the DP nerve), but DE-3’s unique amplitude and shape are discernable in expanded traces (data not shown) and quantified in Results. ***B***, Scatter plot of normalized mean firing rates during the analysis period of US application and in control trials. The mean of each trial in the study is represented as a single point. Thresholds for “excitatory” and “inhibitory” traces are 20% above and below baseline mean, as denoted by the thinner horizontal lines. Mean baseline firing rates ranged from 1.0 to 12.1 Hz (regular US condition) and 1.6 to 10.6 Hz (control condition).

Trial data were excluded from analyses if baseline tonic firing was <1 Hz, or if baseline firing was inconsistent (coefficient of variability >1); paradigms in which trials were excluded, and final sample sizes, are specified in Results. Mean spike frequencies during the peak response and recovery periods were normalized to 30 s baseline means for comparison across trials. Averaged responses across trials are reported as percent change from baseline firing rate ± SE. Nerves were considered responsive if firing rate during the response period differed from baseline by >20%, the benchmark that encompassed most of the variability in firing in control nerves (Fig. 3*B*).

All statistical tests were performed in MATLAB with the exception of power analyses, which were performed with G*Power 3.1 (Erdfelder et al., 2009). All tests assumed α = 0.05. Categorical data were analyzed with Fisher’s exact tests. Hypothesis tests were two-tailed Welch’s *t* tests (parametric data) or Kruskal-Wallis tests (non-parametric data). Continuous data were tested for normality with Shapiro–Wilk tests. Correlations are reported as Pearson’s *r*. *R*^2^ values were obtained with linear regression (least-squares fit). Statistical tests are summarized in Table 1, and are referenced by letters denoting each test described in Results.

### US

We applied 960-kHz US to DP nerves between the ganglion and the suction electrode recording site at the distal end of the DP nerve (Fig. 1*D*). US was generated with a Sonic Concepts H-102MR transducer coupled with a focusing cone filled with degassed, deionized water. Waveforms were designed by an Agilent 33500B Series function generator and triggered by TTL pulses generated by an Axon CNS Digidata 1440A via pClamp software. Waveforms were amplified by an E&I 100W RF linear power amplifier (model 2100L), and impedance matched with a Sonic Concepts matching network. US pulses consisted of 290 cycles and were 300 μs in duration. We applied 500 pulses/s at a 1-kHz pulse repetition frequency; pulse parameters are diagrammed in Figure 2*A*.

Transducer output was characterized by hydrophone (ONDA HNR-0500), as described previously (Collins and Mesce, 2020). Vertical and horizontal cross-sections of linearly interpolated hydrophone measurements at peak amplitudes overlaid with scaled preparation dimensions are shown in Figure 1*E*.

Absolute peak negative pressure was ∼660 kPa, yielding a spatial peak pulse average intensity (I_SPPA_) of 14.52 W/cm^2^. Our 30% duty cycle yielded a spatial peak temporal average intensity (I_SPTA_) of 4.84 W/cm^2^. The transducer was attached to a micromanipulator and positioned such that its peak output was aligned with the center of the ∼5-mm-long DP nerve. The transducer was tilted at a 20° angle from vertical to reduce the potential for generation of standing waves.

### Heat measurement and apparatuses

For heat-only experiments, we used two methods of heat application, a 50-mW laser (650 nm, Visual Fault Locator, J-Deal TL532) with a fiber optic cable attachment (SIMPLEX OS1-9, 125 μm in diameter), and a coiled nickel-chromium wire device made in-house and powered by an adjustable direct current source.

In all experiments, the DP nerve was surrounded on all sides by saline. Because of the thinness of the nerve (∼50 μm in diameter) and the close similarity of the specific heats and thermal conductivities of water and nervous tissue (Elwassif et al., 2006), we approximated nerve heating by measuring local saline temperature increases with a thermocouple (National Instruments model NIUSB-TC01) positioned underneath (in contact with) the DP nerve. Stimuli were applied as described for US and heat-only experiments. Thermocouple measurements were taken at a 1-Hz sampling rate and logged with NCBI thermologger software; data were imported into MATLAB (MathWorks) for plotting and analysis.

### Filtering

US application sometimes caused high-frequency artifact in DP recordings. The amplitude of the artifact was highly variable and was not always resolved or ameliorated with the addition of a bath ground. A digital low-pass Butterworth filter (sampling frequency = 10 kHz; cutoff frequency = 1000 Hz; sixth order) was effective in reducing high frequency artifact (Fig. 2*B*); for consistency, this filter was applied to all traces regardless of stimulus. Beyond high-frequency noise, US application onset and offset were sometimes associated with large-amplitude low-frequency baseline distortions. Affected traces were high pass filtered with a digital Butterworth filter (sampling rate = 10 kHz; cutoff frequency = 200 kHz) to smooth the affected baseline. Residual high-amplitude artifacts were digitally flattened before spike detection to avoid interference; this resulted in a small loss of information (0.5% in noisiest trace). All digital filtering was performed in MATLAB using “butter” and “filter” functions. In addition to filtering, we inverted extracellular traces for more intuitive viewing (the initial vertical deflection from baseline, corresponding to the rising phase of the action potential was made positive).

## Results

### The single-cell approach

Each of the 21 segmental ganglia of the medicinal leech, *H. verbana*, contains a pair of DE-3 motoneurons; each soma is positioned laterally on the dorsal surface of its home ganglion. The DE-3 axon exits each ganglion via the contralateral DP nerve, and its spike is the largest spontaneously active unit in the extracellular DP recording (Ort et al., 1974; Puhl and Mesce, 2008). Importantly, its spontaneous firing property allowed us to examine US’s effects on spontaneous versus evoked activity. A diagram of the leech nervous system and an individual ganglion are shown in Figure 1*A* alongside a Neurobiotin fill of DE-3 (Fig. 1*B*), and representative intracellular and extracellular traces (Fig. 1*C*). The amplitude of the intracellular somatic spike is smaller than a typical axonal action potential because of attenuation (via electrotonic spread) from the distal spike-initiating zone; the somata of invertebrate neurons typically have a low density of voltage-gated ion channels (Melinek and Muller, 1996; Stuart, 1970).

### US modulates the activity of motoneuron DE-3

We characterized the effects of 30-s US applications after determining that this stimulus duration yielded more reliable outcomes than shorter durations (see Materials and Methods). To determine the quantitative effects of 30 s of US on DE-3, we measured the activity of 48 DP nerves (*N* = 48) from 18 leeches. Twenty-six nerves were exposed to 30 s of 960-kHz US; a schematic of an experimental trial is shown in Figure 2. The remaining nerves (*N* = 22) served as untreated controls. Six nerves (four treated with US, two controls) were excluded from analysis because of low spontaneous firing rates (<1 Hz; *N* = 3 from US group, *N* = 1 from control group) or high firing variability (e.g., bursting activity; *N* = 1 each from US and control; for exclusion criteria, see Materials and Methods). Thus, *N* = 22 nerves exposed to US were subsequently analyzed. Maximal changes in DE-3 firing occurred during the last 10 s of application and continued for an additional 10 s (Fig. 2*B*, analysis box in third trace). Firing rates during this 20-s peak period were normalized to the mean baseline firing rate. Representative traces of US-induced inhibitory and excitatory effects are shown in Figure 3*A* alongside a representative control trial. Normalized means of US-treated and control nerve firing rates during the analysis period are displayed in Figure 3*B*. Mean deviation from baseline of firing of all US-treated nerves was 47.3 ± 7.89% (statistics refer to mean ± SEM). Mean deviation from baseline of control nerves was 4.56 ± 5.10%. In control nerves, firing during the analysis window was largely consistent with baseline, with only 2/20 (10.0%) having mean firing rates that differed by >20%. For the US condition, 18/22 (81.8%) of treated nerves showed substantial modulation of activity (Fisher’s exact test, *p* = 2.8518e-06)^a^. In the US group, we observed mainly inhibitory responses (13 out of 18; mean = 43.3 ± 7.63% decrease in firing rate from baseline). There were a few excitatory cases (four out of 18; mean = 60.7 ± 15.1% increase in firing); 1/18 omitted (see Materials and Methods). Some of these excitatory cases in the treated nerves may have been because of some inherent variability across preparations since a similar extent of excitation was also observed across the control nerves. As will be addressed in the following sections, greater excitatory effects may be elicited through US stimulation of presynaptic or synaptic mechanisms rather than direct activation of the soma or axon of a motoneuron; thus, we cannot rule out the possibility that US stimulation was not completely isolated to the DP nerve for the excitatory cases shown in Figure 3*B*.

Finally, as multiple DP nerves were harvested from the same animal, we performed a one-way ANOVA to determine whether normalized mean firing rate during the analysis period was affected by animal. Animal variability did not significantly affect normalized mean firing rate during the response period (*F*_(9,12)_ = 0.7406, *p* = 0.6686)^b^, nor did it affect the mean absolute deviation of DE-3 firing from baseline during this period (*F*_(9,12)_ = 2.2830, *p* = 0.0918)^c^.

We also assessed whether the direction of modulation, or the magnitude of modulation, was affected by baseline firing rate. Normalized mean firing rate during the analysis period did not significantly correlate with baseline firing rate (Pearson’s correlation, *r* = 0.2801, *p* = 0.2067)^d^. However, when we tested for correlation between absolute deviation from baseline during the analysis period and baseline firing rate, we found a marginally significant correlation (Pearson’s correlation, *r* = −0.4261, *p* = 0.0480)^e^, indicating that cells with lower baseline firing rates tended to have greater deviations (either positive or negative) from baseline as a result of US application.

### The effects on motoneuron DE-3 are direct and persist during synaptic isolation

**Figure 4.**
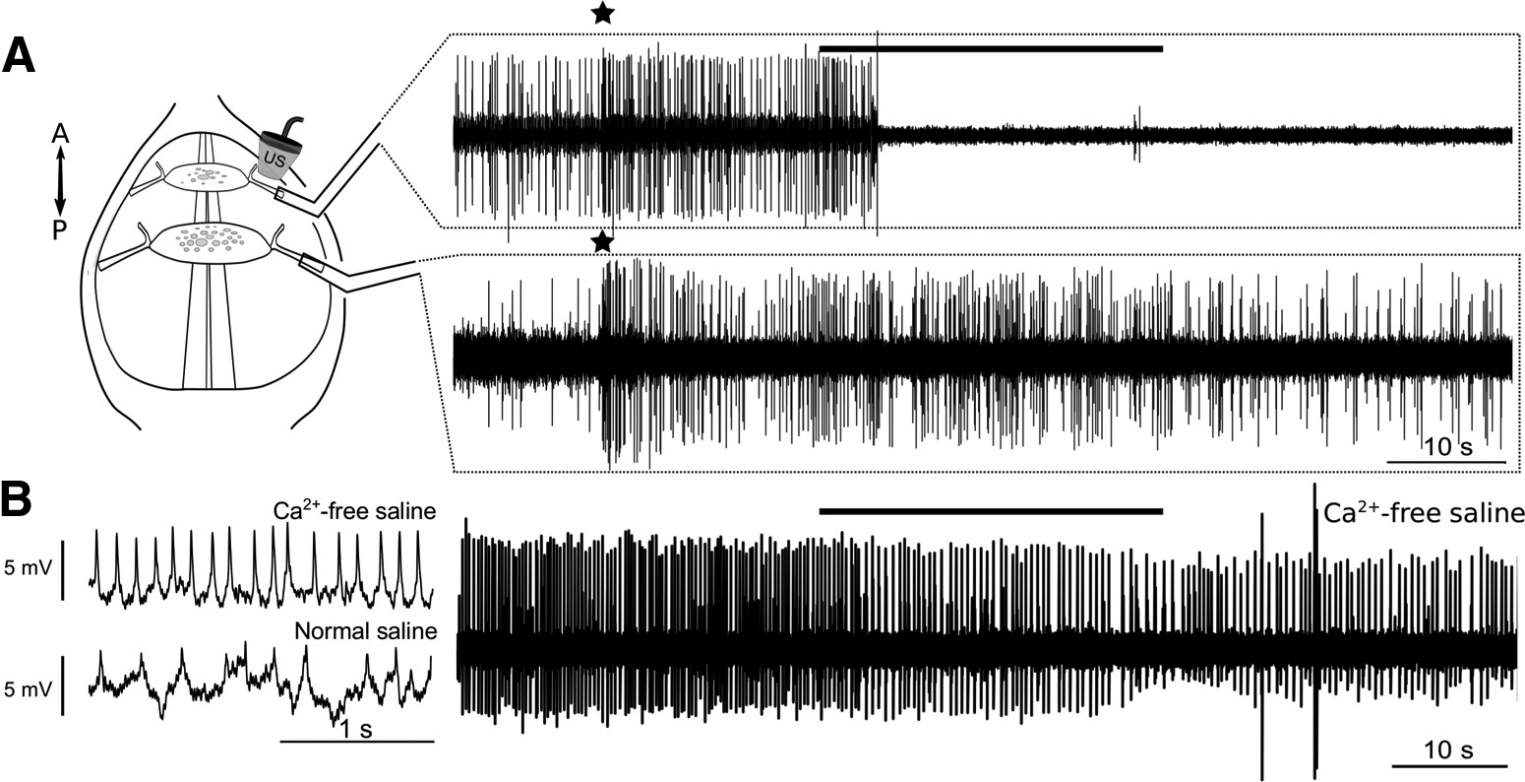
Experiments testing whether US affects the excitability of DE-3 locally and directly. ***A***, Schematic of experimental preparation wherein dual extracellular DE-3 recordings were made; dorsal side is up and the anterior-posterior orientation is marked by double-headed arrow. Because the DE-3 motoneurons (and other cells) in adjacent ganglia often receive common synaptic inputs (note: stars indicate an example of shared response), we tested whether US applied to a DE-3 axon in one DP nerve would similarly affect DE-3 and other units in the segmentally adjacent DP nerve (diagram depicting dual DP recordings, left). Dual extracellular recordings from the DP nerves (right) indicate that US inhibition is limited to the DE-3 targeted (upper trace). None of the nerves responding to US (three of four nerves tested) showed a mirrored effect in the adjacent DP nerve. ***B***, Intracellular recordings of spontaneous DE-3 activity in Ca^2+^-free saline (left, top) and normal saline (bottom, left), showing the reduction of postsynaptic potentials in the absence of Ca^2+^. Blocking synaptic activity (via bathing in Ca^2+^ free saline) does not prevent US from inhibiting DE-3 activity (right trace). Of the seven DP nerves responding to US (*N* = 10; one excluded because of bursting), six (85%) showed an inhibitory response.

To determine whether US effects were specific to the targeted nerve, a subset (*N* = 4) of nerves tested were accompanied by simultaneous extracellular recordings of DP nerves from adjacent ganglia. DE-3 neurons in neighboring ganglia receive common synaptic inputs, and frequently have similar firing patterns. Three out of four tested nerves responded to US and no comparable effects were observable in the neighboring nerves (Fig. 4*A*, simultaneously recorded traces), suggesting US effects were limited to targeted tissue.

To determine whether observed US actions on DE-3 were direct, or a consequence of activation of synaptically-coupled neurons that may have mechanosensitive properties, a subset of US-treated nerves (*N* = 10) were bathed in calcium-free saline. Calcium was replaced with equimolar manganese, which has been shown to block synaptic transmission in the leech, and which produces less rhythmic oscillatory activity than other replacement divalent cations (Angstadt and Friesen, 1991). This loss of synaptic activity is evidenced by the loss of postsynaptic potentials in intracellular DE-3 recordings (Fig. 4*B*). Rhythmic firing was observed in one of the ten nerves before US application, and the trial was aborted (final *N* = 9). A representative trace of US-induced inhibition in Ca^2+^-free saline is shown in Figure 4*B*. The mean baseline firing rate of DE-3 did not differ between conditions of normal saline and Ca^2+^-free saline (3.42 ± 1.33 Hz; for normal saline: 4.56 ± 0.57 Hz; *p* = 0.2164, Wilcoxon rank-sum test)^f^. We observed both excitatory (*N* = 1) and inhibitory (*N* = 6) responses to US within this subset, and a response rate (7/9 nerves, or 77.8%) matching our overall US sample shown in Figure 3*B*, suggesting US’s effects on DE-3 persist in the absence of synaptic input. Importantly, we observed relatively more inhibition in this condition in comparison to the paradigm using normal saline (summary, Fig. 9), suggesting that some of the excitation we observed in the normal saline condition may have been because of the activation of other neurons, perhaps afferents, that travel in the same DP nerve as does DE-3. The single excitatory case in the Ca^2+^-free saline is consistent with the potential outlier cases we observed in control nerve experiments; thus, US applied to DE-3 without synaptic input achieves inhibition of firing activity, which is evident beyond spontaneous fluctuations.

### Heat mimics US’s effects on DE-3

To determine the magnitude of US-associated tissue heating, we placed a thermocouple directly beneath and in contact with the DP nerve to measure changes in temperature during US application. US induced a temperature increase of 3.42 ± 0.12°C (*n* = 3 thermocouple recordings).

**Figure 5.**
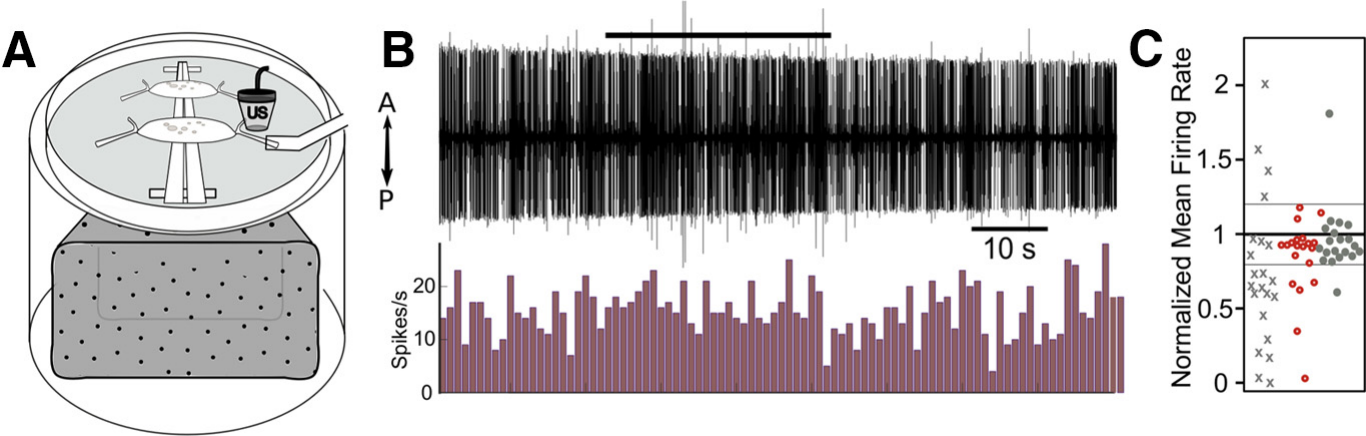
US does not typically modulate neuronal activity in a low-heat paradigm. ***A***, Schematic diagram demonstrating the placement of the latex-bottomed dish placed over a water reservoir filled with sponges. Note: ganglia under investigation in this paradigm have been isolated from the body of the leech (see Materials and Methods). Schematic is not shown to scale; reservoir is ∼10 cm in depth. Double-headed arrow indicates anterior-posterior orientation; dorsal side is up. ***B***, Representative extracellular trace of DE-3 firing during 30 s of US (bar) using the latex dish paradigm (upper). Corresponding histogram of spike frequency (lower; bars = 1-s bins). ***C***, Individual mean firing rates of all nerves in low heat US trials during the analysis period (pink circles) shown alongside results from all nerves in regular US trials (gray Xs) and control trials (gray circles). Mean baseline firing rates ranged from 1.0 to 12.1 Hz (regular US condition), 1.6 to 10.6 Hz (control condition), and 3.3 to 16.0 Hz (latex dish condition).

Recognizing this increase in nerve temperature could be driving the inhibitory effects, we attempted to minimize the preparation’s heating to determine whether effects persisted. We found that our wax substrate contributed to heating by minimizing thermal dissipation. We thus performed US trials on an additional 21 nerves (*N* = 21) on a latex substrate with the recording dish positioned over a large water bath to enable better dissipation of heat (schematic, Fig. 5*A*). One nerve was excluded from analysis because of high variability in baseline-firing rate (final *N* = 20). With this paradigm, the temperature increase was limited to 0.3°C. By greatly reducing heat in this manner, we reduced US modulation (Fig. 5*B*). Only five of 20 (25%) DE-3 motoneurons demonstrated more than a 20% change in firing rate during US application (all inhibited; mean inhibition = 50.9 ± 5.99%; Fig. 5*C*). Although the number of affected nerves did not differ significantly from control (Fisher’s exact test, *p* = 0.436)^g^, a subset of nerves remained susceptible to US modulation despite minimal heating.

We attempted to control further for potential differences associated with our use of different substrates. Standing waves can occur when US reflects off a reflective surface in the direction of the transducer; reflective surfaces are those with a higher acoustic impedance than the surrounding medium, such as our transition from saline to wax. Reflected and emitted waves can summate, causing localized areas of heightened heat and pressure, which have been shown to impact neuronal responsiveness to US by increasing localized radiation force (Menz et al., 2019). Although we had attempted to control for the formation of standing waves by heavily pocking the wax substrate and angling the transducer, as has been shown to greatly reduce the neuromodulatory effects of standing waves (Menz et al., 2019), they nevertheless remained a possibility. To ensure our effects with the higher heat paradigm did not stem in part from higher pressures than those used in the lower heat, non-reflective latex dish paradigm, we doubled US absolute peak negative pressure to 1.3 MPa in four nerves in our low-heat latex dish paradigm. None of the four nerves responded to US, suggesting the purely mechanical effects of US at this frequency, if present, were subtle as compared with thermal effects.

**Figure 6.**
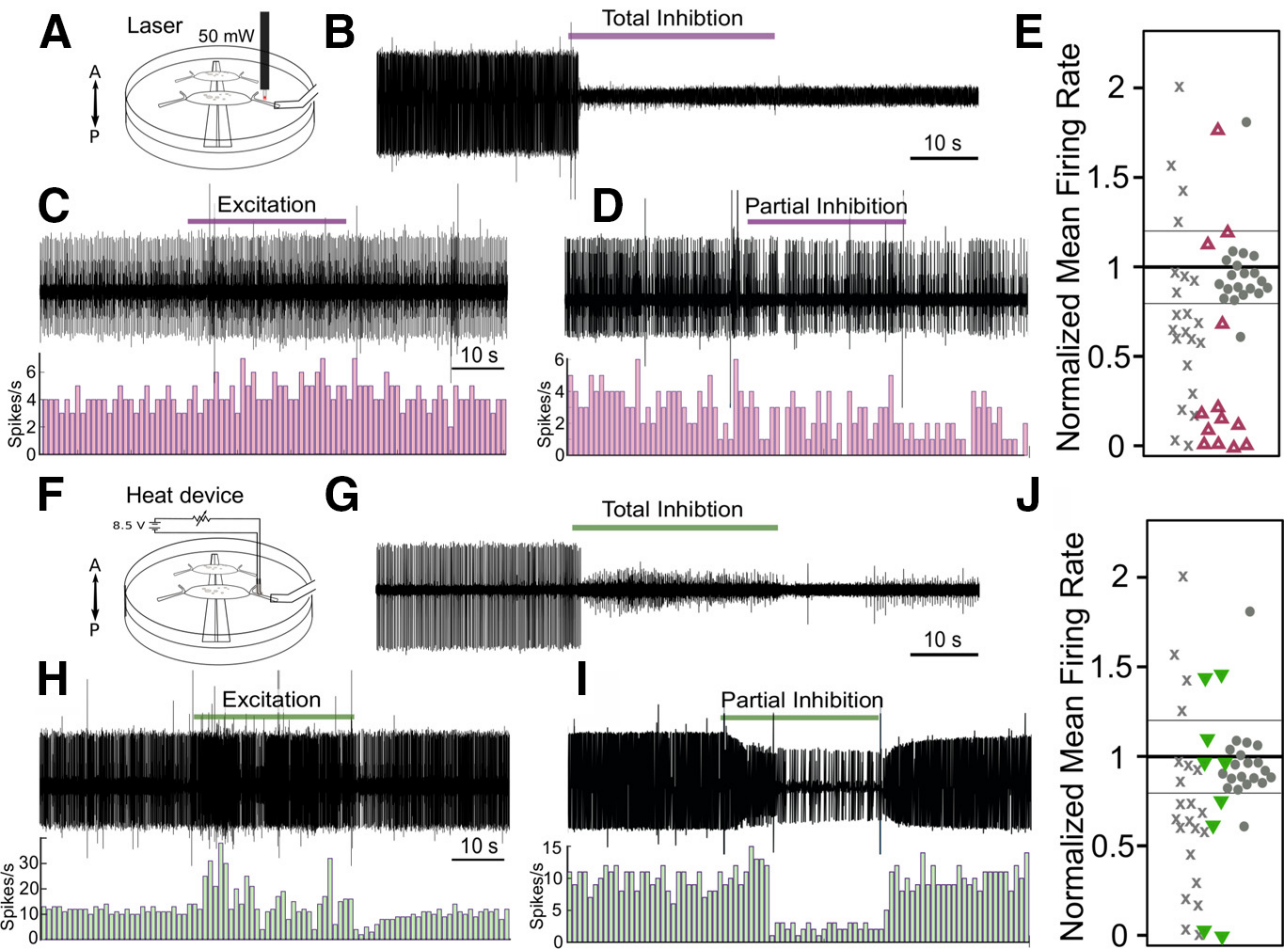
The effects of US can be mimicked by localized application of US-comparable heat. ***A***, ***F***, Schematics of the laser tool and nichrome wire heating device shown respectively for heat application to the DP nerve. Orientation of ganglia are shown by double-head arrows. ***B***, Representative extracellular trace of DE-3 firing with 30 s (bar) of thermal stimulation using the laser (50 mW), resulting in total inhibition, the most frequent outcome. As with US, we also observed some excitation (***C***) and partial inhibition (***D***). ***E***, Individual mean firing rates of all nerves in laser trials during the analysis period (pink triangles) shown alongside results from all nerves in US trials (gray Xs) and control trials (gray circles). Mean baseline firing rates ranged from 1.0 to 12.1 Hz (regular US condition), 1.6 to 10.6 Hz (control condition), and 1.6 to 21.6 Hz (laser condition). Similar results were obtained using the wire device, with representative traces showing predominantly total inhibition (***G***), excitation (***H***), and partial inhibition (***I***). ***J***, Individual mean firing rates of all nerves in wire device trials during the analysis period (green triangles) shown alongside results from all nerves in US trials (gray Xs) and control trials (gray circles). Mean baseline firing rates ranged from 1.0 to 12.1 Hz (regular US condition), 1.6 to 10.6 Hz (control condition), and 6.0 to 15.0 Hz (wire device condition).

We next attempted to replicate the actions of US by inducing comparable US temperature increases in the DP nerve. We found that we could reliably induce a 2.10 ± 0.017°C (n= 3) maximum heat increase in the media surrounding the DP nerve by aiming a 50-mW laser (with a fiber optic attachment) at the nerve for 30 s at the typical site of US application (schematic, Fig. 6*A*). We applied the laser to 14 DP nerves from six animals (*N* = 14). One nerve was excluded from analysis because of its high variability in firing rate (final *N* = 13). Of these nerves, 12 (92.3%) had mean firing rates that differed >20% from baseline during the 30-s laser application period (Fig. 6*E*). The laser produced a faster rate of heating than US; peak effects were observed 10 s into the stimulation and persisted until the end of heat application. Thus, the analysis window was shifted to include data collected during this period (20 s, equivalent to US and control analysis windows). Ten out of 12 responsive DE-3 motoneurons had decreased activity; this inhibition was dramatic (mean = 91.7 ± 6.48%). Two out of 12 were excited (mean = 50.3 ± 14.21% increase in firing). Representative traces of neuromodulatory effects are shown in Figure 6*B–D*.

To ensure this laser-induced inhibition stemmed from heating versus a photic mechanism, we performed additional experiments with an alternative heating mechanism: a small insulated nickel-chromium (nichrome) wire coil connected to a direct current source positioned in the typical location of US application (schematic, Fig. 6*F*). Using the wire heating device, the maximum heat increase of the DP nerve was 4.86 ± 0.064°C (*N* = 3). We tested nine nerves with 30-s applications of heat. As with the laser, the wire heated more quickly than US, and we thus again shifted the analysis window to 40–60 s from trial onset to reflect peak effects. We found that 6/9 (67%) DE-3 motoneurons had mean firing rates that differed from mean baseline rates by >20% (Fig. 6*J*). Four of six DE-3 motoneurons were inhibited and half of these were completely suppressed (mean inhibition 85.7 ± 8.0%). The remaining two modulated nerves were excited; mean excitation = 29.6 ± 8.98%. Representative traces of the effects of the wire are shown in Figure 6*G–I*.

**Figure 7.**
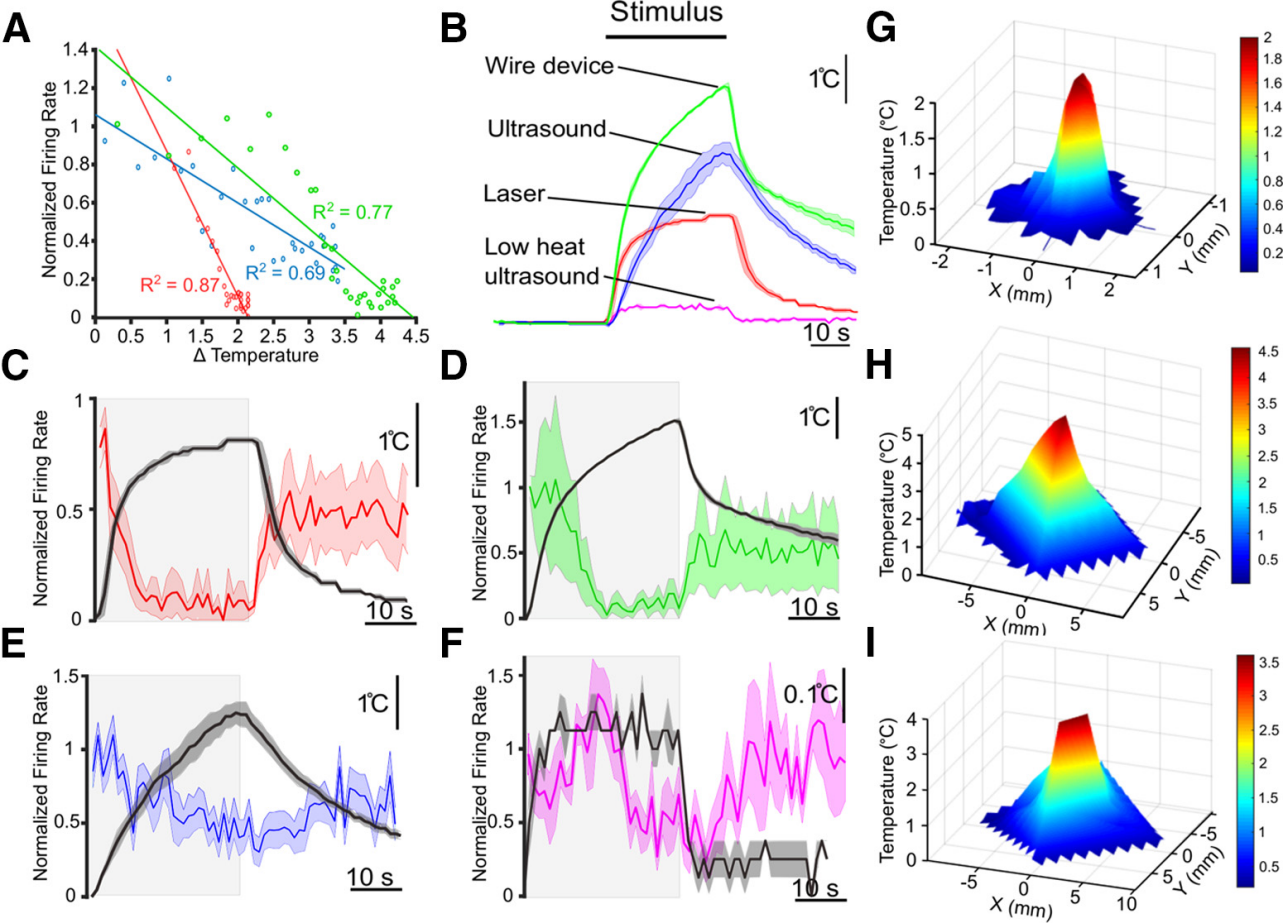
The effects of US can be mimicked by localized application of heat. ***A***, Normalized mean firing rates across inhibitory trials (US, laser, wire device) plotted against the corresponding increase in temperature. ***B***, Averaged thermocouple recordings (*N* = 3) for each stimulus type; central line = mean, shaded areas = ± SEM. ***C–F***, Averaged normalized firing rates across inhibitory trials. Shaded areas = SEM. Stimulus was applied during gray window. Thermocouple recordings are overlaid (mean = black line, gray shaded area = SEM). ***G–I***, Plots of spatial distribution of heat generated by thermocouple recordings of different stimuli in *x* and *y* directions from center (position of nerve). Plots are linearly interpolated from measurements (mean of 2) taken at ¼ mm increments (***G***), or 1 mm increments (***H***, ***I***); stimuli were attached to a notched micromanipulator to ensure accurate movement, thermocouple remained fixed**. *G***, Spatial distribution of heating generated by the laser**. *H***, Spatial distribution of heating generated by the wire device. ***I***, Spatial distribution of heating generated by US.

In total, we observed both inhibition and excitation in response to our three stimuli, with a predominance of inhibitory cases. Stimuli ranged in temperature changes from 2.1°C to 4.9°C. In Figure 7*A*, we plotted the firing rates of inhibitory trials for each stimulus that was averaged across trials against increases in temperature, and found a strong correlation for the US, laser, and wire trials (linear regression, least-squares fit, *R*^2^ = 0.69, 0.87, and 0.77, respectively). With respect to the low-heat US trials, the correlation between the mean firing rates of inhibitory trials and heating was low (*R*^2^ = 0.11). The inhibition observed in these trials may have been because of natural variability in firing versus modulation; the baseline mean coefficient of variability in these trials (0.510 ± 0.047) was slightly higher than in the other US trials (0.425 ± 0.049), although this difference was not significant (*p* = 0.222, Welch’s *t* test)^h^.

### Thermal neuromodulation may be influenced by the spatial spread of heating

Counter to expectations, the stimulus that generated the smallest temperature increase, the laser, produced the most profound inhibition. While the laser had a sharper rate of heat increase than US, this rate was comparable to that of the wire (Fig. 7*C*,*F*); thus, the magnitude of modulation observed with the laser could not be attributed to the rate of heating alone. We thus investigated whether the area of tissue heated differed between the two types of thermal stimuli. To do so, we measured heat increases from a fixed thermocouple at incremental distances in the *x* and *y* directions for all three stimuli. Interpolated plots depicting the spatial spread of heating for each stimulus are shown in Figure 7*G–I*. While the wire and US had similar heating profiles, with peak heating occurring within a 5-mm radius from the center, the laser produced much more focused heating, with peak heating limited to a 1-mm radius from the center. This restricted heating may have accounted for the relatively greater and less reversible inhibition observed with the laser as compared with the other stimuli. Results by stimulus are summarized in Figure 9.

### Poststimulus recovery of US and heat

Recovery from US and heat application was variable. The firing rates of 14/18 (77.8%) US-modulated DE-3 neurons returned to within 20% of baseline, a value consistent with variations in firing in our control nerves. Recovery typically occurred quickly (mean time to recovery = 21.6 ± 16.9 s following the end of stimulation, or ∼10 s after the end of the peak effect period). Excited nerves (*N* = 4) recovered more slowly than inhibited nerves (*N* = 10; 29.3 ± 13.5 vs 18.6 ± 3.70 s). Of the four nerves (all inhibited) that did not return to within 20% of baseline firing, 2/4 partially recovered (50.0% and 74.1% recovery). The remaining two nerves maintained greatly reduced firing rates for the remainder of the nerve’s viability, with one case reaching a maximum of 29.0% of baseline firing rate 106 s after the end of the stimulus period, and the other case firing a single time 60 s after the end of the stimulus. These two minimally recovered nerves were also the most inhibited by US, with 95.1% inhibition and 100% inhibition, respectively.

Recovery rates for heat-only stimuli were similar, with 8/12 (66.7%) modulated nerves treated with the laser and 4/6 (66.7%) nerves treated with the wire returning to within 20% of baseline firing rate. Mean time to recovery with the laser was 18.8 ± 8.63 s (15.0 ± 33.5 s for excited nerves, 22.7 ± 8.66 s for inhibited nerves), and 2.75 ± 1.43 s (4.50 ± 3.50 s for excited nerves, 1.00 ± 0.00 s for inhibited nerves) with the wire. As we observed with US, all nerves that failed to recover fully from heat application had been significantly inhibited (laser: 4/12 nerves, mean inhibition = 99.6 ± 0.0041%; wire: 2/6 nerves, mean inhibition = 97.2 ± 0.025%). Three out of four irreversibly suppressed nerves treated with the laser failed to fire at all poststimulus, as did one of the two nerves irreversibly suppressed with the wire; the other nerves occasionally spiked at rates far below baseline. All nerves that failed to recover were strongly inhibited by stimuli; however, not all strongly inhibited nerves failed to recover. Two nerves whose firing was completely suppressed (100%) by the laser fully recovered, suggesting total suppression need not be irreversible. Differences in recovery rates may have been because of subtle differences in the placement of the stimulus with respect to the nerve, or other stochastic factors beyond the scope of the present study.

### Heat induces conduction block in motoneuron DE-3

**Figure 8.**
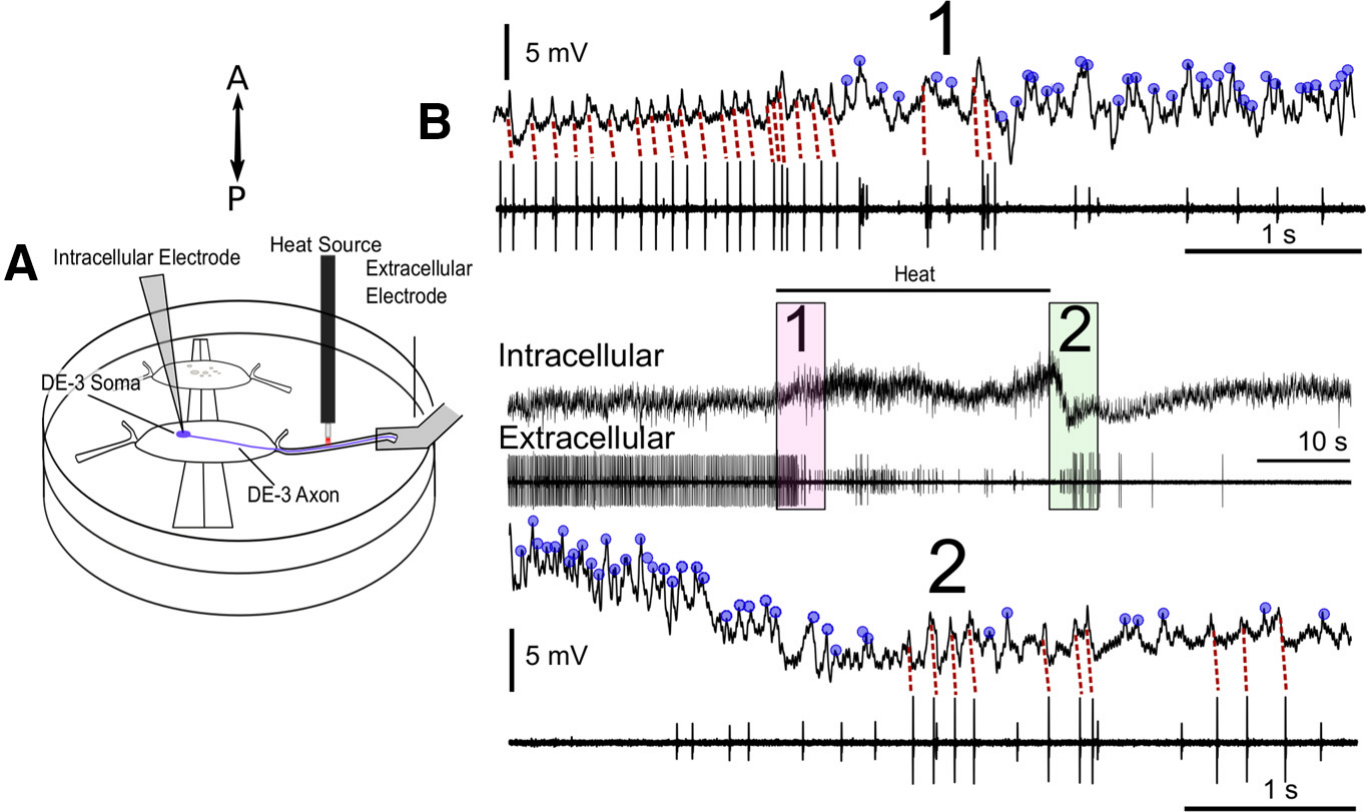
US-comparable heat blocks propagation of the DE-3 spike in the DP nerve. ***A***, Schematic showing the placement of heat delivery (laser) and the position of the dual intracellular and extracellular DE-3 recording sites during heat application (red boxes). Double-head arrow indicates orientation of preparation. ***B***, At the start of the laser heat application (denoted by horizontal bar), the intracellular spike recorded in the soma of DE-3 (near the spike initiation zone) can be seen to correlate one-for-one with the extracellular DE-3 spike (pink inset 1, expansion of first 5 s of stimulus). Upon heat delivery, however, the extracellular spike disappears despite the continuation of the intracellular spike (marked with blue dot), indicating a conduction block at the site of heat application between the spike initiation site and the distal nerve (see later portion of pink-inset expansion, top traces). After termination of heat delivery (green inset 2, expansion of 5 s immediately following the end of the stimulus; bottom traces), partial recovery of the DE-3 axonal spike can be seen. Note (in bottom traces) that waveforms in the intracellular recording are similar whether they are associated with or without their associated axonal spikes (red dotted lines vs blue dots), indicating that we had correctly identified the intracellular activity. In all experiments conducted (*N* = 3), during heat application, the intracellular spike continued in the absence of the mostly silenced axonal spike, which partially recovered after the heat was discontinued.

To determine whether the inhibitory effects of US were because of a broad hyperpolarization of DE-3, or from a local conduction block at the site of stimulus application, we performed intracellular somatic recordings of DE-3 in conjunction with application of the laser placed distally on the DP nerve (*N* = 3). The laser was the most compact heat apparatus, and the most compatible with our intracellular electrode placement. Heat was applied between the somatic intracellular electrode and the distal suction electrode (schematic, Fig. 8*A*). DE-3 activity could thus be measured on either side of the heat stimulus. Figure 8*B* shows a representative simultaneous intracellular and extracellular recording of the DE-3 motoneuron with an inhibitory response with laser stimulation. Spikes initiated near the soma as measured via our intracellular electrode failed to propagate to the distal electrode because of a presumed conduction block at the site of heat application.

### Local versus global heating biases the neuromodulation outcome

**Figure 9.**
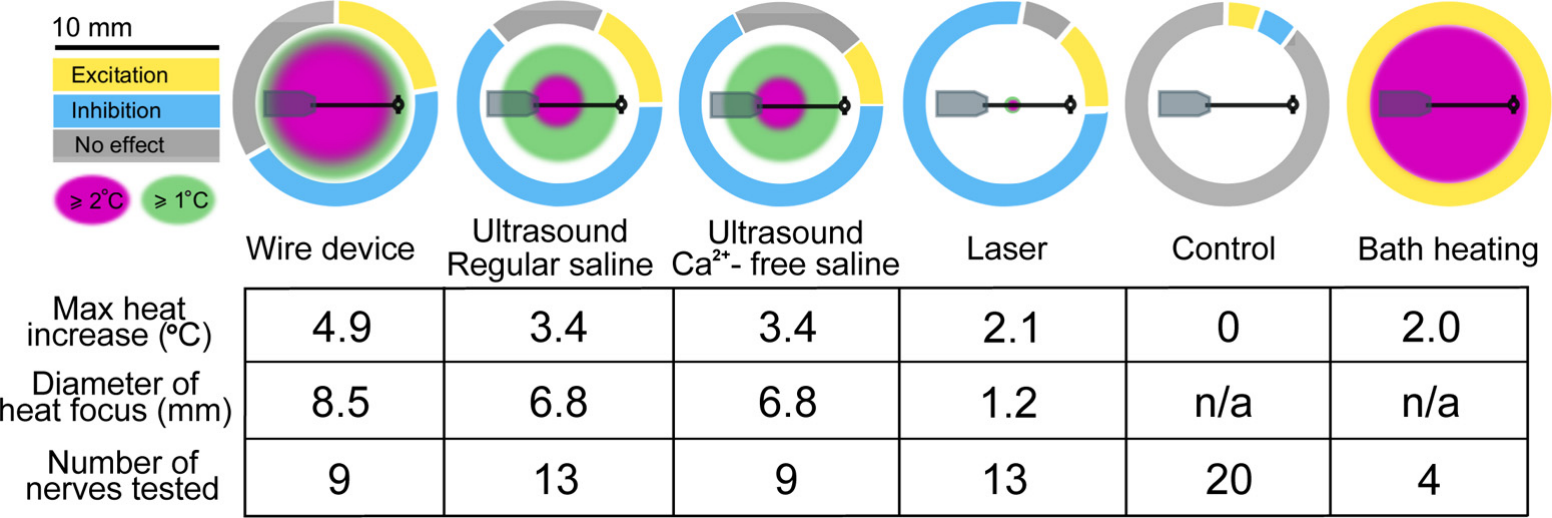
Neuromodulatory actions by stimulus mode. Neuromodulatory effects were found to differ with respect to the spatial properties of stimuli. We observed a trend whereby a broader heat application (e.g., wire device, bath) elicited proportionally more excitation, whereas a narrower heating, including the most focused heat source (i.e., the laser), produced more inhibition. US applied in the Ca^2+^-free condition (blockage of synaptic communication) elicited relatively more neuronal inhibition compared with applications in regular saline. While the thermal properties in this condition were identical to those in the regular saline one, we observed less excitation, which was likely because of the reduction of network-level synaptic inputs that might increase neuronal activity.

To determine whether a global temperature shift of a comparable magnitude over a similar time course (several seconds) could inhibit firing to the extent of focal heating, we raised the bath temperature by 2°C through the rapid addition of heated saline. We found a moderate and short-lived increase in DE-3 firing associated with the addition of heated saline in the four nerves tested (*N* = 4). This effect was comparable to excitatory effects observed in similar bath-heating experiments performed with this preparation (Romanenko et al., 2014). We thus propose that non-noxious thermal inhibitory neuromodulation is only achievable with focused applications of heat, as summarized in Figure 9 based on the combined results presented across our different US and heating experiments.

## Discussion

### Brief overview

In this study, we examined the effects of 30 s of pulsed 960-kHz US on the axon of motoneuron DE-3, a uniquely identified cell in the medicinal leech. Experiments revealed that the primary effect of US, at these parameters, was suppression of neuronal firing via action potential conduction block. A benefit of our study was that response-type variability (i.e., excitatory vs inhibitory) was confined to the same identified neuron, enabling us to avoid confounding results stemming from any inconsistent access to the same or similar types of neurons across recording sessions, which has been problematic in other invertebrate and mammalian studies. Furthermore, by chemically removing synaptic inputs, we could determine whether stimulus-induced outcomes were a function of direct actions on the targeted motoneuron (Fig. 4).

In contrast to achieving both US-induced and US-comparable heat-induced neuronal inhibition, neuronal excitation was difficult to achieve and deemed more likely dependent on synaptic inputs that were indirectly affected, consistent with previous studies performed in intact brain preparations from mammalian species (Guo et al., 2018; Sato et al., 2018).

### Support for a thermal mechanism of US action

Our data support the idea that US modulates motoneuronal activity via a predominantly, if not entirely, thermal mechanism. We arrived at this conclusion in two ways. First, we were unable to modulate DE-3 neuronal activity reliably in the absence of heat (Fig. 5). Second, we performed additional experiments with a 50-mW laser and a wire device, which mimicked US-associated nerve heating, and found that they could reliably mimic the effects of US (Fig. 6). Results from prior studies examining other types of neurons have come to similar conclusions that the heat component of US drives inhibitory responses (Lele, 1963; Ueda et al., 1977; Darrow et al., 2019).

Short applications of US (100 ms, 3.16 s), which did not generate significant heating, failed to inhibit or evoke activity. Furthermore, after significantly reducing US-associated heat from our longer applications (30 s) from 3.5°C to 0.3°C with a less insulating dish substrate, the rate of neuronal inhibition was reduced substantially from 14/22 nerves to 5/20 nerves. These five remaining recordings may have reflected natural variation in firing, as their mean firing rate in inhibitory trials failed to correlate with changes in temperature (*R*^2^ = 0.11), as compared with the heat-only and higher heat US paradigms (*R*^2^ = 0.82, 0.87, and 0.77, for higher heat US, laser, and wire, respectively).

It is noteworthy that we observed the least amount of neuronal excitation in trials performed in Ca^2+^-free saline (1/9 nerves) and with the laser (1/13 nerves). Ca^2+^-free saline prevented sensory cells or other tissues from synaptically exciting DE-3. The laser yielded the most spatially restricted heating, thus limiting the contributions of other neural pathways that may have provided excitatory inputs to the motoneuron as compared with when less focused heat stimuli were used (laser vs wire). These data suggest that thermal excitation stems largely or entirely from circuit-level heating, while targeted axonal heating results in inhibition (summarized in Fig. 9). These conclusions are further supported by our inability to generate neuronal inhibition via bath heating of 2°C, as previously reported in the leech (Romanenko et al., 2014). Finally, the rate and magnitude of inhibition observed, across the different stimuli used, likely stem from differences in the rates of heating (Fig. 7). The rise rate of tissue heating is a salient determinant of neuromodulation outcomes in other forms of thermal neuromodulation, including infrared (Shapiro et al., 2012).

### Consideration of non-thermal components of US on DE-3 activity

Previous studies have proposed that non-thermal mechanisms underlie changes in US-induced neuronal excitation or inhibition, such as intramembrane cavitation (Plaksin et al., 2014) or other mechanical effects, including activation of mechanosensitive ion channels, especially in mechanosensory neurons (Kubanek et al., 2018), and radiation force. Several studies have reported that US at frequencies <400 kHz more efficiently evoke activity than US at higher frequencies (King et al., 2013; Kim et al., 2014). This discrepancy may be driven by a cavitation-based mechanism, as lower frequencies generate cavitational forces more effectively (Gaertner, 1954). We opted to use higher frequencies (960 kHz) to permit more precise targeting of DE-3 (Carovac et al., 2011). This frequency, however, may be too high to generate cavitational actions. In a comparable preparation (invertebrate nerve), cavitation-evoked potentials could be achieved at 0.67 kHz but not 1.1. MHz (Wright et al., 2017). However, given its potential to rupture neuronal membranes (Wright et al., 2017), it remains unclear whether this is a desirable application mode to pursue.

Another factor contributing to our inability to evoke activity mechanically may have been the 660-kPa peak pressure we used. This amount of pressure, however, was significantly higher than levels used in studies of cortical brain neurons, which have attributed US effects to mechanical forces (for example, Tyler et al., 2008; Tufail et al., 2010). Furthermore, it is unlikely that we overshot an effective range of pressures because studies reporting mechanically-attributed transcranial effects of US have covered a range of pressures encompassing ours (0.03–1.11 MPa), and although there is a saturation point with increasing amplitude, there is no associated decline in US responses (King et al., 2013).

Transcranial US stimulation typically involves the modulation of neuronal somata, whereas, in our study, we targeted a neuron’s axon within a peripheral nerve. Thus, one must still consider whether we failed to use a sufficiently high pressure in the context of peripheral nerve activation. This distinction is an important one to consider because peripheral nerves are believed to have higher US activation thresholds than central neural tissues (Wright et al., 2017). Some recent studies of peripheral nerves have revealed that high pressures, far in excess of ours, are needed to evoke motor-related responses; for example, 2 MPa in invertebrates (Wright et al., 2017), and up to 5.4 MPa (Downs et al., 2018), 11.8 MPa (Kim et al., 2020), and 30 MPa (Lee et al., 2020) in mammals. Thus, it may be possible to evoke mechanically-induced effects with higher US pressures. However, pressures in the 10s of MPa range generate intensities that far exceed current safety thresholds for diagnostic use (FDA, 2019), and are likely too destructive for use in reversible neuromodulatory therapies. For reference, the temperature increases in our study (<5°C) have been shown to be safe in mammalian systems for brain-exposure durations ≤60 min (Haveman et al., 2005). In addition, it is encouraging that 100% of the nerves we treated with US remained capable of transmitting DE-3 action potentials, with 78% returning to baseline firing rates within 20 s of stimulus cessation.

The mechanisms underlying thermal inhibition below the range of temperatures known to cause protein degeneration or necrosis (∼45°C in humans, or ∼8°C above normal; Wang et al., 2014) are not completely understood, but may include changes in ion-channel-gating kinetics and conductances. We investigated how US inhibited neural activity thermally at relatively low temperatures (<5°C). As a proxy for US-associated heat, we used the laser, as it was the most compatible with our intracellular recording electrode, and did not generate electrode resonance as does US, which can obscure the fidelity of intracellular recording data (Collins and Mesce, 2020). During heat application, we observed a continuation of spikes recorded in the soma with a loss of spikes distal to the stimulus (Fig. 8), indicating that the inhibition was because of a failure of spike conduction.

### Potential thermal-mediated mechanisms underlying DE-3 inhibition

One promising mechanism to explain the heat-mediated conduction block we observed is a loss of ion homeostasis. It has been shown that thermal suppression of neural activity is accompanied by a spike in extracellular potassium in invertebrate (Money et al., 2009) and mammalian (Wu and Fisher, 2000) systems. At the circuit level, increased [K^+^]_O_ is believed to underlie spreading depression (Kraio and Nicholson, 1978; Somjen, 2001; Ayata and Lauritzen, 2015), a conserved phenomenon in which neural activity is disrupted until concentration gradients are restored (Spong et al., 2016). Importantly, two earlier studies in rat brain found that US can induce spreading depression, resulting in effects reminiscent of pharmacologically raising extracellular potassium (Koroleva et al., 1986) and increasing temperature (Ueda et al., 1977). Spreading depression-associated inhibition can also be preceded by depolarization of the resting membrane potential (Pietrobon and Moskowitz, 2014), and by hyperexcitation (Rodgers et al., 2007). This mechanism thus might explain the brief uptick in firing rate that preceded some of our inhibitory trials, particularly those using the wire (the “hottest” stimulus in the present study), as evidenced by an initial increase in mean firing rate (Fig. 6).

One source of increased [K^+^]_O_ may be an increased conductance through voltage-gated potassium channels (K_V_). In *Aplysia*, heat-mediated (infrared) conduction block is greatly reduced by tetraethylammonium (TEA), a K_V_ antagonist (Ganguly et al., 2019a,b). An additional primary or complementary source of [K^+^]_O_ may be via two-pore potassium channels, which are thermosensitive (Schneider et al., 2014), and whose conductance increases on exposure to US (Kubanek et al., 2016) by a reportedly thermal mechanism (Prieto et al., 2020). Importantly, both classes of potassium channels are expressed ubiquitously by neurons, and a mechanism targeting these channels would circumvent the need to limit US-based neuromodulation therapies to classes of cells that express ion channels susceptible to US mechanical activation, including Piezo (Prieto et al., 2018), TRP (Yoo et al., 2020), and DEG/ENaC/ASIC (Kubanek et al., 2018), or to introduce non-endogenous mechanosensitive ion channels in desired target tissue *a la* sonogenetics (Ibsen et al., 2015). Furthermore, given safety constraints associated with high pressures that may otherwise be required to modulate non-sensory neurons mechanically, thermal applications may be more practical and versatile than mechanical ones.

### Clinical applications

The ability to suppress neuronal activity safely and reversibly could have a significant clinical impact on a wide range of neurologic disorders. The relevance of the results of this study to human health applications is somewhat tempered by inherent differences between mammalian and invertebrate nervous systems, perhaps the most significant of which is the lack of myelination of invertebrate axons. Despite this key difference, action potential conduction in the leech, which was proposed to be thermally inhibited in this study, is governed by the same classes of ion channels that conduct action potentials in mammals, including a rising phase mediated by voltage-gated sodium channels, and a falling phase mediated by K_V_s (Kleinhaus, 1976; Kleinhaus and Prichard, 1976). The distribution pattens of these ion channels, across myelinated mammalian nerves and invertebrate fibers, could result in varying outcomes. However, our results are clearly relevant to the modulation of C-fibers involved in the transmission of pain (Costigan and Woolf, 2000), which like invertebrate nerves are unmyelinated. Based on the results of our study, thermal US may be an effective treatment, not only for pain, but for managing excessive peripheral nerve activity, including peripheral neuropathies (St. John Smith, 2018) and spasticity (Raghavan, 2018).

**Table 1:** Summary of statistical tests

	Data structure	Type of test	Result	Effect size	Power
a	Categorical (binomial)	Fisher’s exact test	*p* = 2.8518e-06	Odds ratio = 40.5	95% CI [6.57,249.65]
b	Leech ID: categorical (nominal) Mean normalized firing rate during response period: normally distributed; *W*_(21)_ = 0.9469; *p* = 0.2353	One-way ANOVA	*F*_(9,12)_ = 0.7406, *p* = 0.6686	*η*^2^ = 0.3571	0.1091
c	Leech ID: categorical (nominal) Normalized mean absolute difference from baseline firing rate: normally distributed; *W*_(21)_ = 0.9279; *p* = 0.1109	One-way ANOVA	*F*_(9,12)_ = 2.2830, *p* = 0.0918	*η*^2^ = 0.6313	0.2794
d	Mean baseline firing rate: non-normal; *W*_(21)_ = 0.8446; *p* = 0.0040 Mean normalized firing rate during response period: normally distributed; *W*_(21)_ = 0.9469; *p* = 0.2353	Pearson’s correlation	*p* = 0.2067	*r* = 0.2801	95% CI [−0.1604,0.6276]
e	Mean baseline firing rate: non-normal; *W*_(21)_ = 0.8446; *p* = 0.0040 Normalized mean absolute difference from baseline firing rate: normally distributed; *W*_(21)_ = 0.9279; *p* = 0.1109	Pearson’s correlation	*p* = 0.0408	*r* = −0.4261	95% CI [−0.7186,−0.0055]
f	Mean baseline firing rate in normal saline: normally distributed; *W*_(12)_ = 0.9104; *p* = 0.1856 Mean baseline firing rate in Ca^2+^-free saline: non-normal; *W*_(8)_ = 0.6781; *p* = 0.0024	Wilcoxon rank-sum test	*p* = 0.2164	*r* = 0.2635	0.2100
g	Categorical (binomial)	Fisher’s exact test	*p* = 0.436	Odds ratio = 2.2500	95% CI [0.3874,13.0665]
h	Coefficient of variability of baseline firing rate in high-heat US trials: normally distributed; *W*_(21)_ = 0.9315; *p* = 0.1317 Coefficient of variability of baseline firing rate in low-heat US trials: normally distributed; *W*_(19)_ = 0.9456; *p* = 0.3046	Welch’s *t* test	*t*_(40)_ = 1.2403, *p* = 0.2221	*d* = 0.1343	95% CI [−0.0530,0.2213]

Letters (leftmost column) correspond to statistical tests as reported in Results. The data structure, test type, result, effect size, and statistical power of these tests are described. Where applicable, results of Shapiro–Wilk tests for normality of data are reported under data structure. Effect sizes for Fisher tests are reported as odds ratios. One-way ANOVA effect sizes are reported as *η*^2^, calculated as the between-groups sum of squares divided by the total sum of squares. Effect sizes for Pearson’s correlation are the correlation coefficients. The effect size for the Wilcoxon rank-sum test is calculated as the *z* statistic divided by the square root of the population size, and the effect size of the Welch’s *t* test was calculated as Cohen’s *d* with a correction for small sample sizes as described (Durlak, 2009). When applicable, power was reported as the 95% confidence interval (CI) or statistical power calculated *post hoc* with G*Power.

## Synthesis

Reviewing Editor: Miriam Goodman, Stanford University Decisions are customarily a result of the Reviewing Editor and the peer reviewers coming together and discussing their recommendations until a consensus is reached. When revisions are invited, a fact-based synthesis statement explaining their decision and outlining what is needed to prepare a revision will be listed below. The following reviewer(s) agreed to reveal their identity: Lidia Szczupak, Jan Kubanek.

Both reviewers appreciated the importance of this study and its relevance to efforts to decipher the cellular mechanism(s) of ultrasound neuromodulation. As you appreciate below, the reviewers had a number of specific suggestions for improvement. The general spirit of these recommendations is to make it easier for readers who are unfamiliar with the leech nervous system and with ultrasound neuromodulation to understand your findings and their limitations. In this spirit, please provide orientation markers (indicating the a-p and d-v axis) for all figures showing schematic drawings of the leech or its ganglia. Additionally, please review all of the figure legends to ensure that they tell the reader the data that are being displayed. Since both intracellular and extracellular recordings are used in your study, please use the figure legends to tell readers which traces are derived from intracellular recordings and which ones are extracellular. For all figures, especially Fig. 8 and 9, please indicate the total number of individual recordings that were made under each condition. For publication in eNeuro, we do expect that multiple replicates are collected for each presented condition and prefer that data presentation include representative examples as well as summary/pooled data. With respect to summary data, such as those shown in Fig. 3b, please consider showing the measured firing rates and using estimation statistics to determine the effect size and statistical significance of the effect. Thank you for submitting your study to eNeuro. I hope that you find this feedback productive and look forward to receiving your revision.

Individual reviews:

Reviewer 1

The study investigates the effects of ultrasound on neuronal activity, focusing on a specific motoneuron in the leech nervous system, and analyzes whether the temperature increase produced by ultrasound is responsible for the described neuronal behavior.

The study is highly relevant as ultrasound is being used experimentally and therapeutically, but the actual mechanisms of action are still under the discussion. The ability of concentrating on an identified neuron has major advantages for the proposed study.

While the experiments are reasonably conceived the presentation of the data needs to be improved as detailed:

Line 124 - "...the rising phase of the DE-3 extracellularly recorded action potential was...". Add extracellularly recorded.

Figure 1-

a. Indicate basic anatomical features: anterior/posterior, ganglion, nerves. The general reader will not understand this scheme.

b. Neurobiotin would not stain the neuron by itself and this marker crosses gap junctions and therefore it would have stained many neurons (see Fan et al 2005, J Comp Physiol A 191: 1157-71). A different cell marker must have been used...?

e. A better description of this figure is needed: 0 mm is in reference to...?; "surface of dish" ... which surface?; the ultrasound transducer is the hydrophone?, if so indicated this in the legend.

If panels d and e were oriented similarly (ganglion to the right, suction electrode to the left) it may help the reader to interpret the figure.

The legend points at an "upper graph"... there is only one graph in this panel.

Line 132 - "... consistent with prior leech electrophysiological studies from other labs". References?

Line 176 - refer to Figure 1d.

Line 234 - "DE-3 is a motoneuron positioned bilaterally.." There are two DE-3 cells per ganglion... as stated it seems that there is one DE-3 per ganglion; change the sentence.

Line 236- "via the contralateral dorsal posterior..."; add contralateral

Line 237- the reference should be Ort et al 1974

Line 241- the spike recorded intracellularly at the soma is smaller than a leech spike... comparing with mammals is misleading because vertebrate and invertebrate Na spikes do not differ in amplitude ...

Line 246 - this section is unnecessary long and confusing... either the authors quantify the duration of the US effects properly or the authors indicate that 10 s was the threshold (2/10 nerves responded with an increase in the firing rate, which is the opposite of what the authors finally consider the US effect!!!) and they decided to use 30s. I would indicate this in the Methods section without much discussion.

Line 258 - "additional DP nerves"... additional to what?

Line 261- indicate the firing rate threshold adopted by the authors.

Line 262 - explain what "high firing variability" means.

Line 264 - a quantitative analysis of the dynamics of the US effect is necessary to support the authors decision to make their measurements at a certain time window. This dynamics is informative in general, and not only to support the authors analysis window.

Figure 2 -

a. is the top trace the DP extracellular recording? If so, indicate this; the red dotted lines do not expand what is shown in the upper trace, do they?; the mid and lower traces belong to Methods, not to results as they describe the stimulation pattern.

b. is an example, the authors need to find a way to quantify the dynamics across different DP nerves.

Figure 3 -

a. The lowest trace in panel a suggests an increase in the firing rate; but as shown, it is hard to judge weather all the spikes correspond to the same neuron. In Methods the authors indicate that they identified spikes by a size threshold, which is usually ok with DP nerves; but for readers that are not familiar with the system the trace will evoke doubts. Expand a segment of the trace and show that spikes are of the same shape.

b. In the analysis there are two aspects to address: the degree of firing frequency change and the % of cases that show no effect, inhibition or excitation. I propose that here and in the rest of the study the authors show both the "normalized mean frequency" graph and the % of cases exhibiting the three different outcomes; in this way the data is displayed in the figures and not scattered throughout the text.

Lines 276-278 - 13/18 inhibitory effects and 4/18 excitatory effects... that leaves one case undefined....?

Figure 4 -

a. MNs in the leech display arborizations that are restricted to the ganglion of origin... the authors state in the figure legend that DE-3 neurons from adjacent ganglia are tightly coupled and that is misleading. The event marked by the stars suggests that the neurons are excited by a common input, but note that in the same example the two DE-3s display mostly different activity patterns, indicating that they are NOT tightly coupled.

The result shown in this figure must be quantified as suggested for Figure 3b, the spikes in the lower trace do not display a fix amplitude and therefore it seems important to follow the whole analysis to support the conclusion that DE-3 from adjacent ganglia are not affected. Did the authors analyze anterior and posterior DP nerves? If the authors want to present this data (not central for the main argument) the analysis has to be complete.

b. These results belong to Figure 3 rather than 4, as the experiments address the nature of the local effect rather than the intersegmental effect.

Lines 324-326 - the summary of the results displayed in Figure 4b is not clear. Please express the results as in Figure 3b. Actually the authors can make the case stronger if they combine the analysis in normal saline and in Ca-free saline in one figure.

Figure 5 - quantify as in Figure 3b. This data is central to the authors argument, the authors cannot present it as if it was a marginal detail.

The inset shows that these experiments where done in isolated pairs of ganglia but the text must emphasize the change in experimental design, the general reader may not capture from the drawing this important aspect.

Figure 6 - as requested for the US stimulation, the authors need to quantify the dynamics of the effect because this is central to the authors analysis. In addition, quantify as in Figure 3b.

Line 431 - the quantification of the recovery needs to be plotted as suggested for figure 3b; otherwise the authors force the reader to write down numbers to compare different treatments. A conclusion regarding the reversibility of the treatment seems very important.

Figure 8 - The result this figure seems to be presenting just one case, is that right? In that case the authors need to repeat this experiment and measure firing freq in the intracellular and extracellular recordings.

In addition the authors need to describe how they recognize spikes in the intracellular recordings, especially when the one-to-one correlation with the extracellular recording is not taking place. The stimulation did not only change the firing activity seen in the nerve, but the intracellular activity measured away from the stimulus, in the soma, was markedly changed too... how can the authors be sure that the "peaks" correspond indeed with fully developed spikes at the site of origin? Looking at the example it seems that the heat propagated its effect in both directions and irreversibly.

Reviewer 2

In this interesting study, the authors applied medium-intensity medium-frequency ultrasound to modulate the conduction of action potentials in axons of specific motor neurons in medicinal leech. A total of 106 nerves from many animals were subjected to this procedure, and the study is therefore adequately powered. Moreover, the effects are substantial, leading to about 40% decrease in the average firing rates over the course of several seconds. Such effect magnitude could have direct implications for noninvasive and targeted neuromodulation in the clinics.

The authors discovered that the neuromodulation was based on a thermal effect. Heating by about a 1-5 degC was required to attain the neuroinhibitory effects. This level of heating is considered safe for minute-level periods of time.

Thermal effects (based on medium-frequency ultrasound) compared to mechanical effects do not suffer from this risk of inertial cavitation, and generally have longer duration of action, both of which are desirable in clinical applications.

The evidence for a thermal effects is solid, multi-fold:

1) brief stimuli, which cannot deposit sufficient amount of energy over time, do not elicit these effects

2) a substrate that conducted heat away from the prep faster diminished the effects

3) localized heating with two other forms of energy replicated the suppressive effects

In addition, the findings support the notion that applications of ultrasound to peripheral structures such as nerves generally requires higher intensities for effects to emerge compared to central nervous system structures. A critical review of this literature suggests that the majority of these studies also rest on a thermal effect. This study therefore further strengthens that notion.

The reported effects are clear and the mechanism of action well supported. The study should therefore be published.

I only have several, mostly minor suggestions for further improvement of this well-executed study.

i) Mechanical effects critically rest on elastic ties of a stimulated target with neighboring tissues. When the authors isolated the dorsal posterior nerve, they disrupted these mechanical ties. Without those ties and only surrounded by saline, the axon can no longer be exposed to the mechanical compressions and expansions that ultrasound would induce in a mechanical tissue continuum. In other words, although the reductionist approach to study the responses of a single structure is laudable, it may also have eliminated potential mechanical effects. This strength/caveat should be added as a new text in the Discussion.

ii) The study would benefit from a more comprehensive presentation of the the dynamics of the effects on the firing rates. Several examples are shown, and then a quantification in a defined interval provided in a main Figure 3b.

Showing the mean+-sem traces of the firing rates as a function of time, separately for the cases for which there is an increase and cases for which there is a decrease in firing rates, would provide further information on the involved mechanisms. For instance, the excitatory dynamics may differ from the inhibitory, which would suggest a distinct mechanism involved in excitation.

iii) Thus far, the first portions of the text, at least on a first read, present the study as one that investigates effects of ultrasound on neurons. The study, nonetheless, stimulated neural processes---axons in particular---instead of somata. There is a consensus (see a paragraph above) that stimulation of neural processes requires higher pressures than of somata, presumably due to the differing levels of ion channels expression and possibly also due to the differing mechanical ties that these structures have with surrounding tissues. I suggest to stress this in the early portions of the text and in the Discussion. An additional benefit of taking this route is that the authors free themselves of explaining out a lack of mechanical effects that have often been reported when stimulating central structures that are dense with somata.

iv) Related to the previous point, the Discussion would benefit from additional text that links the study to the many ultrasonic neuromodulation studies performed in the peripheral nervous system. As noted above, the stimuli used in this study and the discovered mechanism appear to align well with that literature.

v) It is now well appreciated that galvanic coupling of an ultrasound transducer with electrophysiological recordings induces notable artifacts in the recordings (which the authors confirmed) that are difficult to be removed even after careful filtering. I believe that this does not change the results because the inhibitory effect appear to prevail (Fig. 2b) even after the ultrasound offset. However, so that any conclusions are not affected by this artifact, I suggest to shrink the analysis window so that it does not overlap with the ultrasound application time.

vi) Certain Results, e.g., “mean = 43.3% +- 7.63% decrease in firing rate from baseline” would benefit from a statistical assessment of significance.

## Author Response

eNeuro eN-NWR-0514-20

The authors are very appreciative of the valuable feedback from the reviewers and the editor.

Below are the point-by-point responses of the authors (not bolded) in response to reviewers’

comments (in bold).

Synthesis of Reviews:

Significance Statement Comments for Author (Required):

Please revise. The significance statement should name the model system used and also

the claim of significance for application to chronic pain (last sentence) is not consistent

with the utility of studying the US response of motorneurons “devoid of sensory inputs”.

Line 29: We have included the scientific name of the animal model used. We also removed the

statement “devoid of sensory inputs” so that we can retain the message that US has relevance

to the treatment of chronic pain, which is salient and discussed in our Discussion section.

Comments on the Visual Abstract for Author (Required):

N/A

Synthesis Statement for Author (Required):

Both reviewers appreciated the importance of this study and its relevance to efforts to

decipher the cellular mechanism(s) of ultrasound neuromodulation. As you appreciate

below, the reviewers had a number of specific suggestions for improvement. The general

spirit of these recommendations is to make it easier for readers who are unfamiliar with

the leech nervous system and with ultrasound neuromodulation to understand your

findings and their limitations. In this spirit, please provide orientation markers (indicating

the a-p and d-v axis) for all figures showing schematic drawings of the leech or its

ganglia. Additionally, please review all of the figure legends to ensure that they tell the

reader the data that are being displayed. Since both intracellular and extracellular

recordings are used in your study, please use the figure legends to tell readers which

traces are derived from intracellular recordings and which ones are extracellular. For all

figures, especially Fig. 8 and 9, please indicate the total number of individual recordings

that were made under each condition. For publication in eNeuro, we do expect that

multiple replicates are collected for each presented condition and prefer that data

presentation include representative examples as well as summary/pooled data. With

respect to summary data, such as those shown in Fig. 3b, please consider showing the

measured firing rates and using estimation statistics to determine the effect size and

statistical significance of the effect. Thank you for submitting your study to eNeuro. I

hope that you find this feedback productive and look forward to receiving your revision.

The individual reviews are included below.

We appreciate the helpful feedback and as a result have provided orientation markers in the

figures; we also include more precise descriptions of which traces are extra- and intracellular

recordings in the legends. For figures, especially Figs. 8 and 9, we now include the total number

of individual recordings for each condition. Because the firing rates are all normalized, it did not 2

make sense to us to indicate or show more absolute firing rates. We have made all the

requested changes to the figures and have added additional analyses unless otherwise noted.

Individual reviews:

Reviewer 1

The study investigates the effects of ultrasound on neuronal activity, focusing on a

specific motoneuron in the leech nervous system, and analyzes whether the temperature

increase produced by ultrasound is responsible for the described neuronal behavior.

The study is highly relevant as ultrasound is being used experimentally and

therapeutically, but the actual mechanisms of action are still under the discussion. The

ability of concentrating on an identified neuron has major advantages for the proposed

study.

While the experiments are reasonably conceived the presentation of the data needs to be

improved as detailed:

Line 124 - “...the rising phase of the DE-3 extracellularly recorded action potential was...”.

Add extracellularly recorded.

The requested change has been made.

Figure 1-

a. Indicate basic anatomical features: anterior/posterior, ganglion, nerves. The general

reader will not understand this scheme.

We thank this reviewer for their helpful comments. To ensure that a reader not familiar with the

leech nervous system is better able to understand its neuroanatomical organization, we have

incorporated all of the suggested additions. We have also included a magnified image of a

segmental ganglion and have rearranged parts of the figure so as to accommodate the

additional anatomical descriptions.

b. Neurobiotin would not stain the neuron by itself and this marker crosses gap junctions

and therefore it would have stained many neurons (see Fan et al 2005, J Comp Physiol A

191: 1157-71). A different cell marker must have been used...?

We are absolutely sure that Neurobiotin was used as the neuronal marker. We have found that

dye coupling with Neurobiotin is capricious, and with all due respect to the reviewer, we often

find that the DE-3 motoneuron is labeled individually or with faintly labeled cells. We selected

the Neurobiotin-filled DE-3 shown in Fig. 1 because it best demonstrated its full morphology.

Note: there is a tiny and faintly labeled soma shown in the figure if you look closely, but it is not

worth mentioning in the manuscript.

e. A better description of this figure is needed: 0 mm is in reference to...?; “surface of

dish” ... which surface?; the ultrasound transducer is the hydrophone?, if so indicated

this in the legend.

If panels d and e were oriented similarly (ganglion to the right, suction electrode to the 3

left) it may help the reader to interpret the figure.

The legend points at an “upper graph”... there is only one graph in this panel.

As requested, we have oriented panels d and e so that they match in their orientation (e.g.,

electrode to the left). We have also described, in the legend, more of the details shown in the

figure, such as the different scales and what they mean, and have better defined existing

terminology.

Line 132 - “... consistent with prior leech electrophysiological studies from other labs”.

References?

To comply with eNeuro’s process of anonymity, we limited references to our own work. We now

provide relevant references.

Line 176 - refer to Figure 1d.

Made the change.

Line 234 - “DE-3 is a motoneuron positioned bilaterally..” There are two DE-3 cells per

ganglion... as stated it seems that there is one DE-3 per ganglion; change the sentence.

Changed to read: Each of the 21 segmental ganglia of the medicinal leech, Hirudo verbana,

contains a pair of DE-3 motoneurons; each soma is positioned laterally on the dorsal surface of

its home ganglion.

Line 236- “via the contralateral dorsal posterior...”; add contralateral

Made the change.

Line 237- the reference should be Ort et al 1974

We have included the Ort reference, thank you.

Line 241- the spike recorded intracellularly at the soma is smaller than a leech spike...

comparing with mammals is misleading because vertebrate and invertebrate Na spikes

do not differ in amplitude ...

Point taken...we replaced the word ‘mammalian’ with ‘axonal’ action potential.

Line 246 - this section is unnecessary long and confusing... either the authors quantify

the duration of the US effects properly or the authors indicate that 10 s was the threshold

(2/10 nerves responded with an increase in the firing rate, which is the opposite of what

the authors finally consider the US effect!!!) and they decided to use 30s. I would indicate

this in the Methods section without much discussion.4

We now make mention of the shorter duration FUS testing in the Methods section. We begin our

results section with, “We characterized the effects of 30 second US applications after

determining that this stimulus duration yielded more reliable outcomes than shorter durations (N

= 10; see Methods).”

Line 258 - “additional DP nerves”... additional to what?

We omitted the word additional

Line 261- indicate the firing rate threshold adopted by the authors.

In the Methods, our stated criterion was < 1Hz; for the sake of clarity we have included that in

line 261.

Line 262 - explain what “high firing variability” means.

In the Methods we stated that ‘high firing variability’ had a coefficient of variability > 1. To

provide an example of variability we have clarified this sentence by inserting “(e.g., bursting

activity)”.

Line 264 - a quantitative analysis of the dynamics of the US effect is necessary to

support the authors decision to make their measurements at a certain time window. This

dynamics is informative in general, and not only to support the authors analysis window.

The analysis window was selected based on a time frame surrounding the maximal amount of

change observed over the population of responses (a quantitative assessment), which in all

cases fell within 20 s after FUS stimulation. For consistency across samples, the window

became a fixed standard to measure the US responses across animals. We explored the

dynamics of responses to US and other thermal stimuli in Fig. 7, which indicated that the rate of

DE-3 change was correlated to the rate in the rise of temperature. Our study establishes that

US has mostly an inhibitory action on the activity of DE-3, which appears to be a consequence

of an increase in temperature. Future studies of the dynamics of DE-3 is relation to additional

parameters in both stimulation modes and response features would likely be informative, in

general, but are beyond the scope of the current study.

Figure 2 -

a. is the top trace the DP extracellular recording? If so, indicate this; the red dotted lines

do not expand what is shown in the upper trace, do they?; the mid and lower traces

belong to Methods, not to results as they describe the stimulation pattern.

b. is an example, the authors need to find a way to quantify the dynamics across different

DP nerves.

We now better describe the details of this Fig. Fig. 2 is, indeed, a mix of Results and Methods,

but we thought it was very effective for readers to see the US-related application parameters in

conjunction with the raw data. 5

The red dots expand only the time scale (not the physiological data), but that way the reader

can see how the recording and the stimulus parameters relate to each other. We have

quantified the peak effects of the stimuli and normalized them with regard to pre-stimulus firing.

Presently, our paper is quite long with many different experiments. Diving deeper into the

temporal dynamics of the effects is beyond the scope of the current study, which examines if the

US is excitatory or inhibitory.

Figure 3 -

a. The lowest trace in panel a suggests an increase in the firing rate; but as shown, it is

hard to judge weather all the spikes correspond to the same neuron. In Methods the

authors indicate that they identified spikes by a size threshold, which is usually ok with

DP nerves; but for readers that are not familiar with the system the trace will evoke

doubts. Expand a segment of the trace and show that spikes are of the same shape.

Unfortunately, expanding the trace ruins the look of the figure and the DE-3 units are already

quantified in the Results. We have included clarification that not all the spikes in the bottom

trace belong to DE-3, but that we can resolve them. “FUS-excited units larger than DE-3 can

obscure the DE-3 spike (which is the largest spontaneous unit in the DP nerve), but DE-3’s

unique amplitude and shape are discernable in expanded traces (data not shown) and are

quantified in the Results.”

b. In the analysis there are two aspects to address: the degree of firing frequency change

and the % of cases that show no effect, inhibition or excitation. I propose that here and in

the rest of the study the authors show both the “normalized mean frequency” graph and

the % of cases exhibiting the three different outcomes; in this way the data is displayed

in the figures and not scattered throughout the text.

Yes, these data are described in the text. In revised Fig. 9, the number of cases is shown.

Lines 276-278 - 13/18 inhibitory effects and 4/18 excitatory effects... that leaves one case

undefined....?

We include that 1/18 was omitted.

Figure 4 -

a. MNs in the leech display arborizations that are restricted to the ganglion of origin... the

authors state in the figure legend that DE-3 neurons from adjacent ganglia are tightly

coupled and that is misleading. The event marked by the stars suggests that the neurons

are excited by a common input, but note that in the same example the two DE-3s display

mostly different activity patterns, indicating that they are NOT tightly coupled.

The result shown in this figure must be quantified as suggested for Figure 3b, the spikes

in the lower trace do not display a fix amplitude and therefore it seems important to

follow the whole analysis to support the conclusion that DE-3 from adjacent ganglia are

not affected. Did the authors analyze anterior and posterior DP nerves? If the authors 6

want to present this data (not central for the main argument) the analysis has to be

complete.

We agree that mention in the legend that the segmentally adjacent DE-3s are “tightly” coupled is

unwarranted; we now mention that the cells share common inputs. As DE-3 does not run

through the anterior nerve we did not analyze that nerve. The goal of the study for Fig. 4 was

simply to determine if FUS stimulation of one DP nerve might have a more global effect on other

cells, including DE-3, which are located beyond the ganglion being stimulated. Clearly, Fig. 4

shows that DE-3 can be robustly inhibited in one ganglion but not in the adjacent ganglion. In no

cases did we find that FUS application caused any similar effects that were mirrored in the two

DP nerves. Even though the reviewer mentions that these data are not central to the main story,

we think that they have value even without a more rigorous analysis. The revised legend now

states, “Because the DE-3 motoneurons (and other cells) in adjacent ganglia often receive

common synaptic inputs (Note: stars indicate an example of shared response), we tested if US

applied to a DE-3 axon in one DP nerve would similarly affect DE-3 and other units in the

segmentally adjacent DP nerve (diagram depicting dual DP recordings, left). Dual

extracellular.... None of the nerves responding to FUS (3 of 4 nerves tested) showed a mirrored

effect in the adjacent DP nerve.”

b. These results belong to Figure 3 rather than 4, as the experiments address the nature

of the local effect rather than the intersegmental effect.

Lines 324-326 - the summary of the results displayed in Figure 4b is not clear. Please

express the results as in Figure 3b. Actually the authors can make the case stronger if

they combine the analysis in normal saline and in Ca-free saline in one figure.

Fig. 3 provides the initial overview of the type of DE-3 responses that we observed with FUS

application. Fig. 4 then dives deeper into whether the FUS responses obtained are a result of

indirect (more global) network activation or can be a result of direct (non-synaptic) actions. The

field of FUS had wrestled with these issues, and thus we wanted to combine these possible

outcomes in one figure. The “intersegmental” effect is a way of looking at whether FUS might

activate common interneurons or sensory cells that then cause the DE-3 changes observed in

one and possibly both adjacent DE-3s. In Fig. 4b, we get to the heart of the debate by blocking

synaptic inputs to DE-3. Importantly, we find that FUS still works to affect DE-3, mostly by

inhibiting DE-3 (in 6 of 7 samples that responded). In lines 324-326 we mentioned the summary

results in Fig. 3b; we did not state Fig. 4b (as 4b has no summary results). So 77% (7 of 9) DP

nerves responded to FUS under synaptic blockade and 85% (6 of 7) showed inhibition.

Figure 5 - quantify as in Figure 3b. This data is central to the authors argument, the

authors cannot present it as if it was a marginal detail.

The inset shows that these experiments where done in isolated pairs of ganglia but the

text must emphasize the change in experimental design, the general reader may not

capture from the drawing this important aspect.

These data in Fig. 5 are now shown plotted in a similar manner as in Fig. 3b. In addition, we

now state, “Note: the ganglia under investigation in this paradigm have been isolated from the

body of the leech (see Methods).” Our previous Methods section and other text (Results) 7

highlight this distinction, but we agree that it will help the reader with such changes incorporated

into the legend.

Figure 6 - as requested for the US stimulation, the authors need to quantify the dynamics

of the effect because this is central to the authors analysis. In addition, quantify as in

Figure 3b.

Line 431 - the quantification of the recovery needs to be plotted as suggested for figure

3b; otherwise the authors force the reader to write down numbers to compare different

treatments. A conclusion regarding the reversibility of the treatment seems very

important.

We have quantified as in Fig. 3b, and associated changes were made in the figure, paper and

legend. In aggregate, the reversibility of treatments is plotted in Fig. 7.

Figure 8 - The result this figure seems to be presenting just one case, is that right? In

that case the authors need to repeat this experiment and measure firing freq in the

intracellular and extracellular recordings.

In addition the authors need to describe how they recognize spikes in the intracellular

recordings, especially when the one-to-one correlation with the extracellular recording is

not taking place. The stimulation did not only change the firing activity seen in the nerve,

but the intracellular activity measured away from the stimulus, in the soma, was

markedly changed too... how can the authors be sure that the “peaks” correspond indeed

with fully developed spikes at the site of origin? Looking at the example it seems that the

heat propagated its effect in both directions and irreversibly.

We apologize that the presentation of these data was unclear. In the legend of Fig. 8, we have

clarified when the heat is turned on and off, and highlight that activity in DE-3 does return. We

also guide the reader to see that similar intracellular waveforms exist when axonal spikes are

present or absent. We now state, “Upon heat delivery, however, the extracellular spike

disappears despite the continuation of the intracellular spike (marked with blue dot), indicating a

conduction block at the site of heat application between the spike initiation site and the distal

nerve (see later portion of pink-inset expansion, top traces). After termination of heat delivery

(green inset 2, expansion of five seconds immediately following the end of the stimulus; bottom

traces), partial recovery of the DE-3 axonal spike can be seen. Note (in bottom traces) that

waveforms in the intracellular recording are similar whether they are associated with or without

their associated axonal spikes (red dotted lines versus blue dots), indicating that we had

correctly identified the intracellular activity. In all experiments conducted (N = 3), during heat

application, the intracellular spike continued in the absence of the mostly silenced axonal spike,

which partially recovered after the heat was discontinued.

Reviewer 2

In this interesting study, the authors applied medium-intensity medium-frequency

ultrasound to modulate the conduction of action potentials in axons of specific motor

neurons in medicinal leech. A total of 106 nerves from many animals were subjected to

this procedure, and the study is therefore adequately powered. Moreover, the effects are 8

substantial, leading to about 40% decrease in the average firing rates over the course of

several seconds. Such effect magnitude could have direct implications for noninvasive

and targeted neuromodulation in the clinics.

The authors discovered that the neuromodulation was based on a thermal effect. Heating

by about a 1-5 degC was required to attain the neuroinhibitory effects. This level of

heating is considered safe for minute-level periods of time.

Thermal effects (based on medium-frequency ultrasound) compared to mechanical

effects do not suffer from this risk of inertial cavitation, and generally have longer

duration of action, both of which are desirable in clinical applications.

The evidence for a thermal effects is solid, multi-fold:

1) brief stimuli, which cannot deposit sufficient amount of energy over time, do not elicit

these effects

2) a substrate that conducted heat away from the prep faster diminished the effects

3) localized heating with two other forms of energy replicated the suppressive effects

In addition, the findings support the notion that applications of ultrasound to peripheral

structures such as nerves generally requires higher intensities for effects to emerge

compared to central nervous system structures. A critical review of this literature

suggests that the majority of these studies also rest on a thermal effect. This study

therefore further strengthens that notion.

The reported effects are clear and the mechanism of action well supported. The study

should therefore be published.

I only have several, mostly minor suggestions for further improvement of this well executed study.

The authors wish to thank this reviewer for their positive feedback and for their affirmation of the

importance of our findings

i) Mechanical effects critically rest on elastic ties of a stimulated target with neighboring

tissues. When the authors isolated the dorsal posterior nerve, they disrupted these

mechanical ties. Without those ties and only surrounded by saline, the axon can no

longer be exposed to the mechanical compressions and expansions that ultrasound

would induce in a mechanical tissue continuum. In other words, although the

reductionist approach to study the responses of a single structure is laudable, it may

also have eliminated potential mechanical effects. This strength/caveat should be added

as a new text in the Discussion.

Thank you for this idea. However, after careful consideration, we can not make the case that the

recorded nerve (in either the intact or isolated preparation) has a possible loss of a ’mechanical

tissue continuum’. The leech’s DP nerve is normally quite stretchy and compensates for the

large changes in body volume as the animal flattens and elongates during swimming or

shortens during crawling. Essentially, when the freed end of the DP nerve is sucked up into the

electrode, no slack remains in the nerve and it is fairly taut (the nerve is also not pulled too

tightly). Essentially, after placement in the suction electrode, the nerve has some tension on it

as if it were tethered to the body wall. 9

ii) The study would benefit from a more comprehensive presentation of the the dynamics

of the effects on the firing rates. Several examples are shown, and then a quantification

in a defined interval provided in a main Figure 3b.

Showing the mean+-sem traces of the firing rates as a function of time, separately for the

cases for which there is an increase and cases for which there is a decrease in firing

rates, would provide further information on the involved mechanisms. For instance, the

excitatory dynamics may differ from the inhibitory, which would suggest a distinct

mechanism involved in excitation.

Thank you for the interest in the dynamics of the firing rates, but tackling such issues is beyond

the scope of the current paper. We have approached such issues and found that such analyses

quickly become very complex. The reason is that each nerve has its own ’personality’, and that

is why we had to normalize the firing activity to baseline activity. Finding trends in excitatory

versus inhibitory preparations are not based on any set of simple metrics, which we have yet to

find. For example, we see US-induced inhibition in preparations that are firing at high rates or

low rates. If we were able to greatly expand our sample size by conducting many more

experiments, trends might emerge, but that would significantly delay publication of the current

story.

iii) Thus far, the first portions of the text, at least on a first read, present the study as one

that investigates effects of ultrasound on neurons. The study, nonetheless, stimulated

neural processes---axons in particular---instead of somata. There is a consensus (see a

paragraph above) that stimulation of neural processes requires higher pressures than of

somata, presumably due to the differing levels of ion channels expression and possibly

also due to the differing mechanical ties that these structures have with surrounding

tissues. I suggest to stress this in the early portions of the text and in the Discussion. An

additional benefit of taking this route is that the authors free themselves of explaining

out a lack of mechanical effects that have often been reported when stimulating central

structures that are dense with somata.

We now clarify in the Introduction that we studied the axon of the DE-3 motoneuron. We have

addressed the central versus peripheral issue in the Discussion (see paragraph below)

iv) Related to the previous point, the Discussion would benefit from additional text that

links the study to the many ultrasonic neuromodulation studies performed in the

peripheral nervous system. As noted above, the stimuli used in this study and the

discovered mechanism appear to align well with that literature.

We have addressed these important points and have included the following text:

Line 571. Transcranial US stimulation typically involves the modulation of neuronal somata,

whereas, in our study, we targeted a neuron’s axon within a peripheral nerve. Thus, one must

still consider whether we failed to utilize a sufficiently high pressure in the context of peripheral

nerve activation. This distinction is an important one to consider because peripheral nerves are

believed to have higher US activation thresholds than central neural tissues (Wright et al.,

2017). Some recent studies of peripheral nerves have revealed that pressures, far in excess of

ours, are needed to evoke motor-related responses; for example, 2 MPa in invertebrates 10

(Wright et al., 2017), and up to 5.4 MPa (Downs et al., 2018), 11.8 MPa (Kim et al., 2020), and

30 MPa (Lee et al., 2020) in mammals. Thus, it may be possible to evoke mechanically-induced

effects with higher US pressures, but at what cost?

v) It is now well appreciated that galvanic coupling of an ultrasound transducer with

electrophysiological recordings induces notable artifacts in the recordings (which the

authors confirmed) that are difficult to be removed even after careful filtering. I believe

that this does not change the results because the inhibitory effect appear to prevail (Fig.

2b) even after the ultrasound offset. However, so that any conclusions are not affected

by this artifact, I suggest to shrink the analysis window so that it does not overlap with

the ultrasound application time.

We appreciate the reviewer’s suggestion of shrinking the analysis window away from the US

application time; however, even though our data might look “cleaner” we feel that by doing so

would limit valuable information occurring with shorter response times. We feel that changes in

DE-3 fire frequency after filtering, during US application, remain a reliable indicator of neuronal

activity.

vi) Certain Results, e.g., “mean = 43.3% +- 7.63% decrease in firing rate from baseline"

would benefit from a statistical assessment of significance.

Because our controls were within the 20th percentile, we did not think that a 100% decrease in

firing rate needed a statistical test
